# Cooperative DNA Recognition Modulated by an Interplay between Protein-Protein Interactions and DNA-Mediated Allostery

**DOI:** 10.1371/journal.pcbi.1004287

**Published:** 2015-06-11

**Authors:** Felipe Merino, Benjamin Bouvier, Vlad Cojocaru

**Affiliations:** 1 Computational Structural Biology Group, Department of Cell and Developmental Biology, Max Planck Institute for Molecular Biomedicine, Münster, Germany; 2 Center for Multiscale Theory and Computation, Westfälische Wilhelms University, Münster, Germany; 3 Bioinformatics: Structures and Interactions, Bases Moléculaires et Structurales des Systèmes Infectieux, Univ. Lyon I/CNRS UMR5086, IBCP, Lyon, France; University of Maryland, UNITED STATES

## Abstract

Highly specific transcriptional regulation depends on the cooperative association of transcription factors into enhanceosomes. Usually, their DNA-binding cooperativity originates from either direct interactions or DNA-mediated allostery. Here, we performed unbiased molecular simulations followed by simulations of protein-DNA unbinding and free energy profiling to study the cooperative DNA recognition by OCT4 and SOX2, key components of enhanceosomes in pluripotent cells. We found that SOX2 influences the orientation and dynamics of the DNA-bound configuration of OCT4. In addition SOX2 modifies the unbinding free energy profiles of both DNA-binding domains of OCT4, the POU specific and POU homeodomain, despite interacting directly only with the first. Thus, we demonstrate that the OCT4-SOX2 cooperativity is modulated by an interplay between protein-protein interactions and DNA-mediated allostery. Further, we estimated the change in OCT4-DNA binding free energy due to the cooperativity with SOX2, observed a good agreement with experimental measurements, and found that SOX2 affects the relative DNA-binding strength of the two OCT4 domains. Based on these findings, we propose that available interaction partners in different biological contexts modulate the DNA exploration routes of multi-domain transcription factors such as OCT4. We consider the OCT4-SOX2 cooperativity as a paradigm of how specificity of transcriptional regulation is achieved through concerted modulation of protein-DNA recognition by different types of interactions.

## Author Summary

Pluripotent stem cells can give rise to all somatic lineages. When taken out of the context of the embryo they can be maintained and for this a core transcriptional regulatory circuitry is crucial. OCT4 and SOX2, two factors of this network, are also critical for the induction of pluripotency in somatic cells. In pluripotent cells, OCT4 and SOX2 associate on DNA regulatory regions, enhancing or modifying each other's sequence specificity. In contrast, in the early stages during induction of pluripotency, it was proposed that OCT4 explores the genome independent of SOX2. Here we report the mechanism by which SOX2 influences the orientation, dynamics, and unbinding free energy profile of OCT4. This involves an interplay of protein-protein interactions and DNA-mediated allostery. We consider that this mechanism enables OCT4 to use its DNA binding domains and the interaction partners available in a certain biological context to access alternative genome exploration routes. This study enhances the understanding of the context specific function of OCT4 and provides a general perspective on how DNA-binding cooperativity is modulated by different types of interactions.

## Introduction

Transcription factors recognize short DNA sequences found in the regulatory regions of genes. In eukaryotic cells, a large number of biologically irrelevant binding sites are present due to the large size of their genomes. In addition, different transcription factors can share DNA specificities, due to homology or convergence. Therefore, the correct choice of gene targets has to rely on a more sophisticated mechanism than pure DNA-binding specificity. To increase gene regulation specificity, regulatory elements contain a high number of tandem transcription factor binding sites, known as enhanceosomes. Notably, only specific combinations of proteins can bind them to control gene expression [1,2]. Due to the close proximity of their binding sites within the enhanceosomes, transcription factors bind cooperatively, modifying their affinities for these particular sites and creating a transcriptional response that is both highly specific and sensitive [1]. Moreover, their protein-protein interactions can evoke latent DNA specificities, causing them to occupy binding sites rarely bound by the isolated protein [3]. Interestingly, transcription factors can even bind cooperatively in the absence of physical interaction between them due to DNA-mediated allostery [4,5,6]. Despite recent advances in understanding the atomic structure of some enhanceosomes [7], the structural details behind enhanceosome assembly are still poorly understood.

Multiple DNA-binding domains can also regulate the specificity and affinity of modular transcription factors through the increase in the length of their sequence specific binding sites [1]. Furthermore, the intrinsic difference in the DNA-binding affinities of the individual domains allows these proteins to employ alternative DNA-recognition mechanisms [8]. For instance, the members of the POU family are characterized by a DNA-binding region composed of two independent DNA-binding domains, a POU specific (POU_S_) and a POU homeodomain (POU_HD_), which are connected by a flexible linker. Both domains contain a helix-turn-helix fold from which one helix docks into the major groove of the DNA, establishing the majority of the sequence specific protein-DNA contacts [9]. In addition, the N-terminal end of the POU_HD_ contains a disordered region that docks into the minor groove and contributes significantly to DNA binding [10]. Combined, the two domains recognize the consensus sequence ATGC(A/T)AAT, where the POU_S_ recognizes the first half and the POU_HD_ the second. The POU factors are involved in the control of a wide variety of biological processes [11]. In particular, the POU factor OCT4 lies in the core of the transcriptional network controlling the maintenance and induction of stem cell pluripotency [10]. Together with other transcription factors in this network, it mediates enhanceosome assembly in pluripotent stem cells [12].

Whole genome chromatin immuno-precipitation (ChIP) analysis in embryonic stem cells have shown that, predominantly, OCT4 binds to DNA in combination with SOX2 (consensus binding site: C(T/A)TTGTT), to a composite motif made by the juxtaposition of their individual binding sites (canonical motif) [13]. They bind cooperatively to this motif forming a protein-protein interaction interface in which only the POU_S_ of OCT4 interacts with SOX2 [14,15]. Moreover, although the OCT4-SOX2 interaction occurs only upon binding to DNA [16], SOX2 assists the *in vivo* DNA recognition process of OCT4 [12]. Interestingly, they also bind cooperatively to composite motifs with different spacing between their individual sites [14]. Experiments with chimeric proteins have shown that depending of the composite motif, either the POU_S_ or the POU_HD_ are the most relevant for binding, suggesting that SOX2 can influence their relative contribution to the binding affinity [17,18]. In addition, ChIP experiments suggest that OCT4 binds to DNA alone during the initial stages of reprogramming to pluripotency [19]. Therefore, OCT4 may employ alternative DNA-recognition mechanisms depending on the cellular and genomic context. Whereas the atomic structure of some POU-SOX complexes and many POU homodimers bound to semi-palindromic sites are known [15,20,21,22], the structural basis for the inter-molecular communication of these proteins is still not understood. Interestingly, nuclear magnetic resonance (NMR) studies have demonstrated that co-binding with SOX2 to the HO*XB1 e*nhancer, which contains a consensus canonical motif, modifies the DNA-binding mechanism of the OCT4 homolog OCT1, by altering the way in which the individual domains scan the DNA [8].

*UTF1* is a key coactivator in pluripotent cells. The enhancer of this gene contains a canonical motif under the control of the OCT4-SOX2 combination [18]. In human, the sequence at the 3' end of the OCT4 binding site (5'-CATTGTTATGCTAGC-3') lacks part of the sequence-specific POU_HD_ binding site, making the binding of the POU_HD_ to this site partly unspecific. Interestingly, the ability to recognize this sequence strongly correlates with the ability to maintain the pluripotent state in stem cells [23], suggesting that the recognition of degenerate sequences is a key component of OCT4's biological function.

Recently we investigated the OCT4-SOX2 interface on an idealized canonical motif by classical molecular dynamics simulations [24]. Whereas in that study we focused on the protein-protein interface, in this work we explore how OCT4 recognizes degenerate binding sites such as that found in the *UTF1* enhancer and the role of SOX2 in this process. For this, we used unbiased molecular dynamics simulations combined with simulations of protein-DNA unbinding and free energy profiling. More generally, we aimed at understanding how DNA-binding cooperativity involving transcription factors with multiple DNA binding domains is modulated. As a result, we provide a mechanism by which the function of such transcription factors may acquire cellular and genomic context specificity.

## Results

### SOX2 modifies the orientation and dynamics of the DNA-bound configuration of OCT4

**Fig 1:**
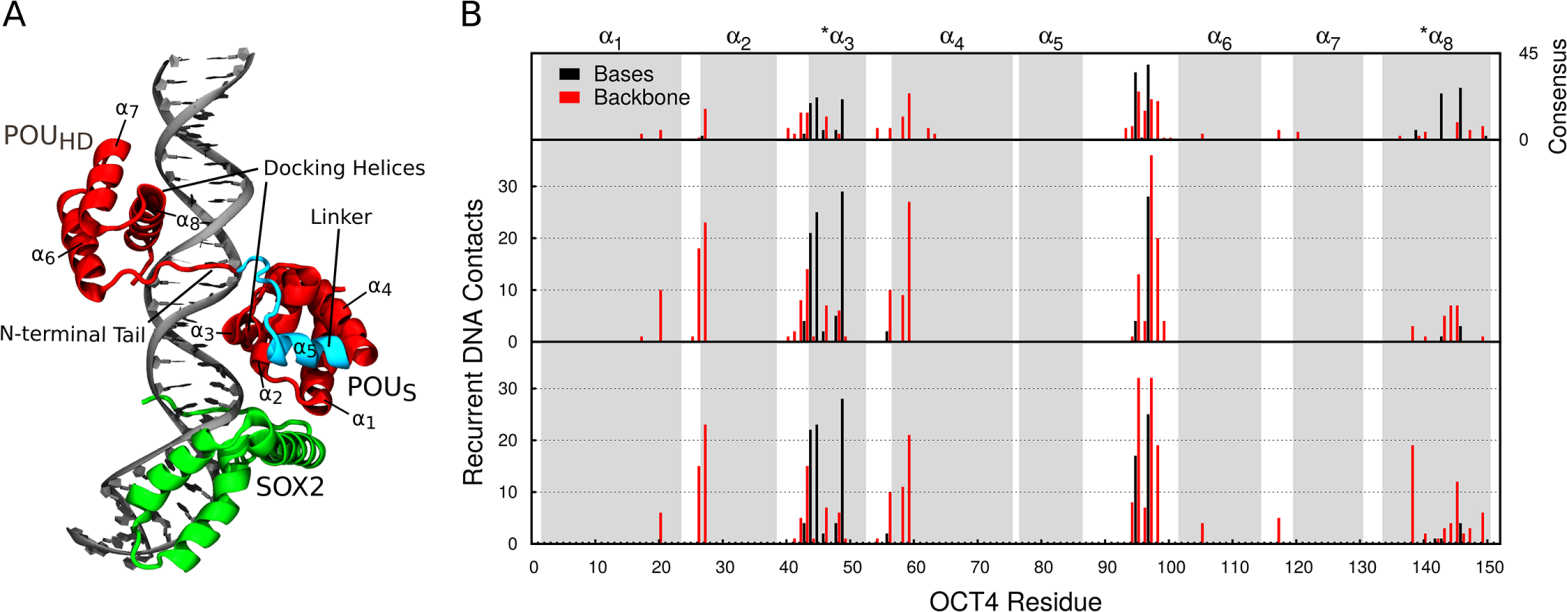
Interactions and structural changes at the protein-DNA interfaces. (A) The OCT4-SOX2-*UTF1* complex. (B) Per-residue number of recurrent protein-DNA contacts. The top plot shows the number of contacts in a model of the OCT4-SOX2-*HOXB1* (consensus sequence) complex as reference. The middle and bottom plots show the data from the unbiased simulations of the OCT4-DNA and OCT4-SOX2-DNA complexes respectively. The gray boxes highlight the 8 helices of OCT4. α_1_ – α_4_ correspond to the POU_S_, α_6_ – α_8_ to the POU_HD_. See also S1 and S2 Figs.

To characterize the OCT4-DNA interfaces we performed 2 ensembles of 1.8 μs of unbiased simulations each, one for the OCT4-*UTF1* complex and one for the OCT4-SOX2-*UTF1* complex (Fig 1A). Each ensemble is composed of 4 independent, 450-ns-long simulations. Unless otherwise specified, all results are derived from the ensemble analysis.

To study the protein-DNA interactions, we created contact maps containing all the atom-atom contacts within a distance threshold of 4.5 Å (S1A Fig). We then defined as recurrent contacts those formed in more than 50% of the total simulation time (S1 Table). In the absence of SOX2, most of the recurrent POU_S_-DNA contacts (S1 Table) cluster around the docking helix, consistent with the orientation of this domain when bound to consensus sequences (Figs 1B and S2A). For the POU_HD_, most of the recurrent contacts involve the N-terminal tail of the domain (S1 Table), mainly due to the insertion of R95 and R97 into the minor groove (Figs 1B and S2A). Notably, although the globular region of the POU_HD_ remains bound to the major groove (S1B Fig), it forms only few recurrent contacts with the DNA (Figs 1B and S2A, S1 Table). In contrast, when OCT4 binds to consensus sequences, the residues V139, N143, and Q146 from the docking helix of the POU_HD_ form sequence-specific contacts with the DNA bases at the 3' end of binding site (top plot in Fig 1B). As these bases are different in the *UTF1* sequence, N143 and Q146 contact the DNA through non-stable interactions with the DNA backbone.

In the presence of SOX2, the OCT4-SOX2 interface is formed, mainly through the hydrophobic contacts between I21 from helix α_1_ of the POU_S_ and the SOX2 residues A61 and M64, as well as some transient electrostatic interactions with the linker region of OCT4 (S2B Fig). These protein-protein contacts have only minor effects on the POU_S_-DNA contact map (Figs 1B and S1A), including a small decrease in the number of contacts of T45 with the DNA bases (Figs 1B, S2B and S2C) and of residues in helices α_1_ and α_4_ with the DNA backbone (Figs 1B, S2B and S2D). Importantly, the POU_S_-DNA interface is similar to the one observed when bound to consensus sequences, irrespective of the presence of SOX2. On the other hand, SOX2 modifies the POU_HD_-DNA contact map (S1A Fig) even in the absence of a direct interaction. SOX2 induces the formation of several recurrent interactions between the POU_HD_ and the DNA backbone both in the tail and globular part of POU_HD_ (Figs 1B and S2, S1 Table). This suggests that an allosteric communication between the POU_S_-SOX2 interface and the POU_HD_ contributes to the OCT4-SOX2 cooperativity.

**Fig 2:**
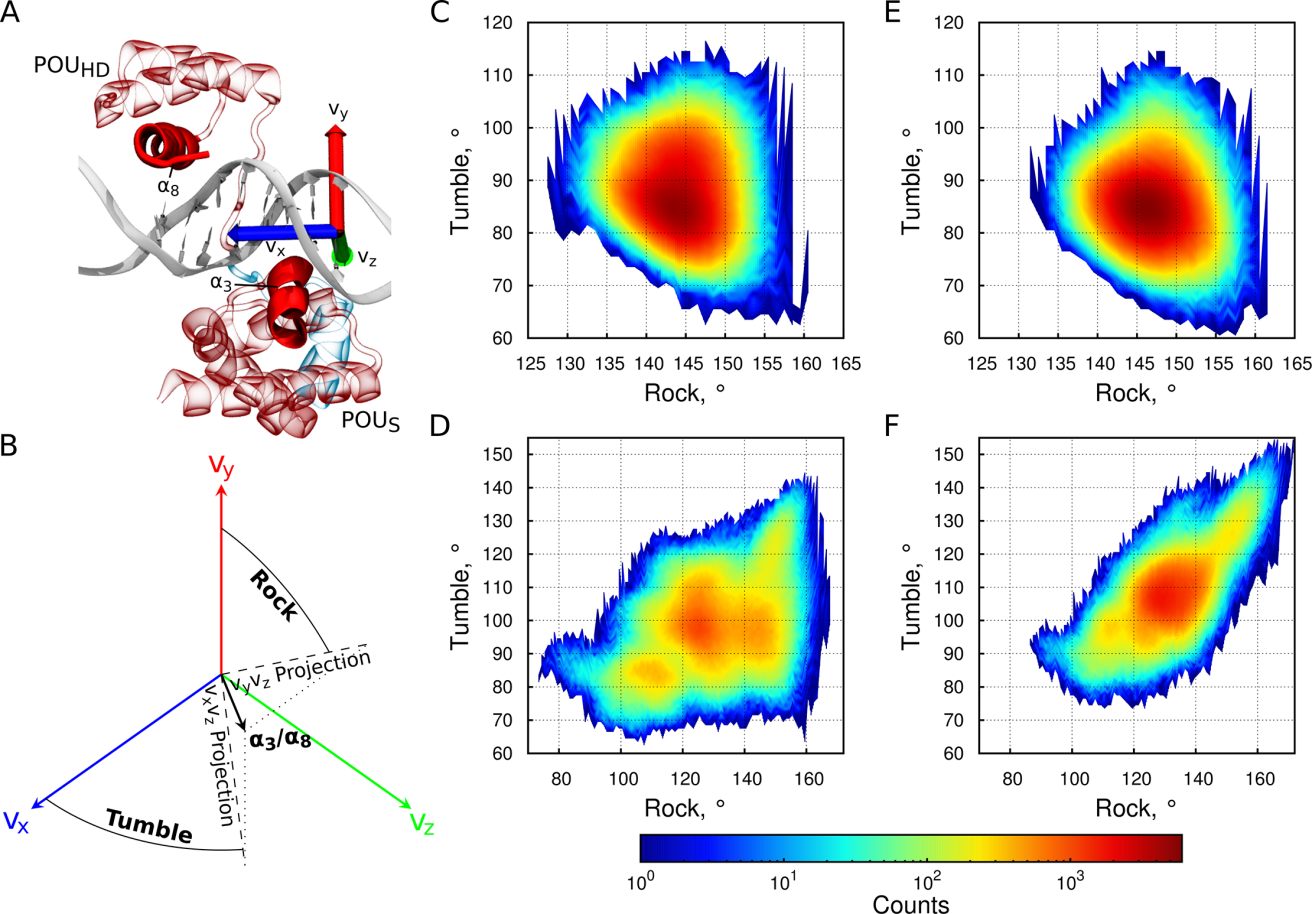
Orientational dynamics in DNA-bound configurations. (A,B) Schematic view of the coordinate system (A) and definition (B) of the Rock and Tumble angles describing the orientation of the docking helices (shown as opaque cartoons) of the two domains of OCT4. Rock-Tumble histograms in the absence (C,D) or presence (E,F) of SOX2 for the POU_S_ (C,E) and POU_HD_ (D,F). See also S3 Fig.

To explore the dynamics of the POU_S_ and POU_HD_ domains relative to their binding sites, we calculated the orientation of the docking helices (Fig 1A) around the helical axis (Rock) and inside the binding groove (Tumble) (Fig 2A and 2B). Consistent with the small number of recurrent POU_HD_-DNA contacts observed in the absence of SOX2 (Fig 1B, S1 Table), the binding orientation of the POU_HD_ fluctuates more than that of the POU_S_ (Fig 2C and 2D). When SOX2 is present, there is a 14% decrease in the fluctuation of the POU_S_ orientation (S2 Table) calculated from the distributions of the Rock and Tumble angles (Fig 2C and 2E). However, the effect of SOX2 on the POU_S_ orientation and dynamics is subtle and further sampling may be necessary for its correct quantification. In addition, SOX2 induces a reorientation and a decrease in the dynamics of the POU_HD_ (Fig 2D and 2F). The diagonal pattern in the Rock versus Tumble histogram (Fig 2F) suggests that SOX2 couples the motion of the POU_HD_ to the major groove of the DNA, reflecting the increased number of protein-DNA interactions of the globular region of the POU_HD_ in the presence of SOX2 (Fig 1B, S1 Table). Importantly, these results were found to be consistent in all individual simulations from the ensembles (S3 Fig).

### SOX2 and OCT4 communicate through DNA-mediated allostery

**Fig 3:**
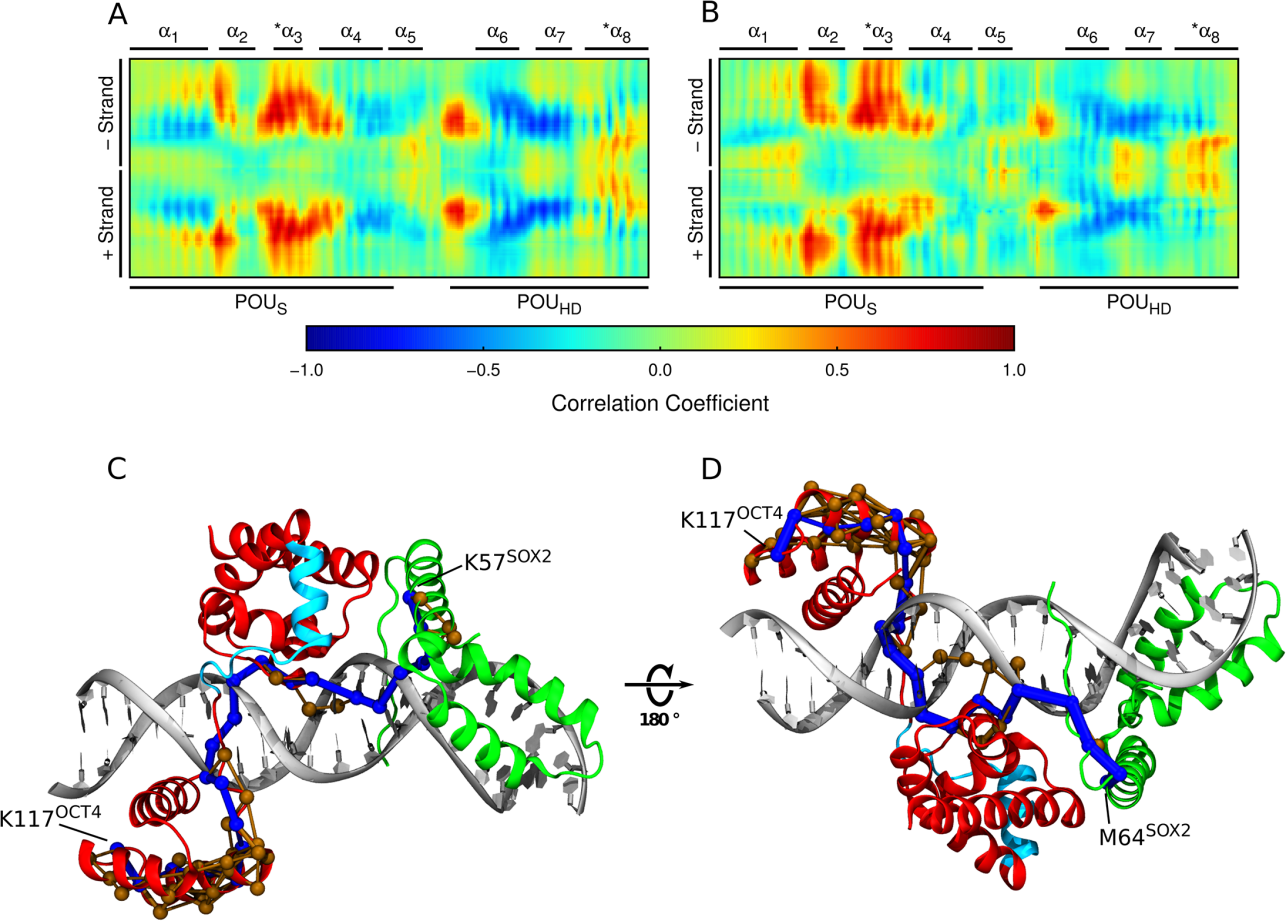
Correlated motions and allosteric communication pathways. (A,B) Positional cross-correlation between OCT4 and the DNA in the absence (A) or presence (B) of SOX2. Each docking helix is marked with a star. The two DNA strands are labeled with “+” and “-”. (C,D) Shortest communication paths between K57 from SOX2 and K117 from OCT4 (C) and between M64 from SOX2 and K117 from OCT4 (D). The size of the edges between nodes is proportional to the number of paths crossing them. The shortest path is shown in blue while the suboptimal paths in light brown. See also S4 Fig.

Our results suggested that the POU_HD_ and the OCT4-SOX2 interface communicate through an allosteric signal. To explore this, we calculated a positional cross-correlation matrix from the simulations. For consistency, we superimposed OCT4 and its binding site in the binary and ternary complexes. In the absence of SOX2, the helices α_2_ and α_3_ of the POU_S_ and the N-terminal tail of the POU_HD_ are correlated with the OCT4-binding site (Fig 3A). In addition, the helices α_6_ and α_7_ of the POU_HD_ are anticorrelated with the POU_S_ binding site, whereas the helix α_8_ was only slightly correlated with its binding site. When SOX2 is present, the correlations of DNA regions with the POU_S_ are extended, whereas those involving the POU_HD_ tail and globular part are diminished (Fig 3B). Notably, this correlation pattern in which the tail of the POU_HD_ correlates with the same DNA region as the POU_S_ rather than the globular region of its own domain suggests that OCT4 may be divided into three units with independent roles in DNA binding.

To elucidate the pathway used to propagate the allosteric signal from the POU_S_-SOX2 interface to the POU_HD_, we performed a network analysis on the trajectories of the ternary complex. For this, a reduced representation of the protein and DNA is generated by defining nodes to represent groups of atoms. Two of these nodes are connected if within the atoms represented by them, an atom-atom pair stays within 4.5 Å for more than 75% of the simulation time. The distance between two connected nodes in this network reflects their positional cross-correlation calculated from the simulation. Finally, optimal signal propagation pathways between two distant nodes are estimated by minimizing their separation distance which is computed by adding the distances between connected nodes along the pathway (see Methods for details). For this, we generated a new cross-correlation matrix, superimposing OCT4, SOX2 and the composite motif. Independent of the end-points, the shortest communication pathways between SOX2 and the POU_HD_ do not cross the SOX2-POU_S_ interface nor the partially structured linker peptide of OCT4, but propagate through either the DNA or the POU_S_-DNA interface (Fig 3C and 3D). The optimal path travels from SOX2 to DNA and then cross the POU_S_-DNA interface and through the DNA reach the tail of the POU_HD_. An alternative path that only threads through the DNA to reach the POU_HD_ tail connects M64^SOX2^ to K117^OCT4^ (Fig 3D). Combined, these results suggest that the allosteric interaction between SOX2 and OCT4 is mediated mainly by changes in the DNA structure induced by their DNA-binding domains. Interestingly, the globular region and the tail of the POU_HD_ belong to different communities (S4 Fig) which are regions of the network wherein the correlation between the nodes is higher than to the rest of the network. This is in agreement with the correlation pattern calculated by superimposing only OCT4 and its binding site (Fig 3A and 3B), adding further evidence that the two regions of the POU_HD_ may function independently in DNA recognition.

### OCT4 and SOX2 modify DNA structural properties

**Fig 4:**
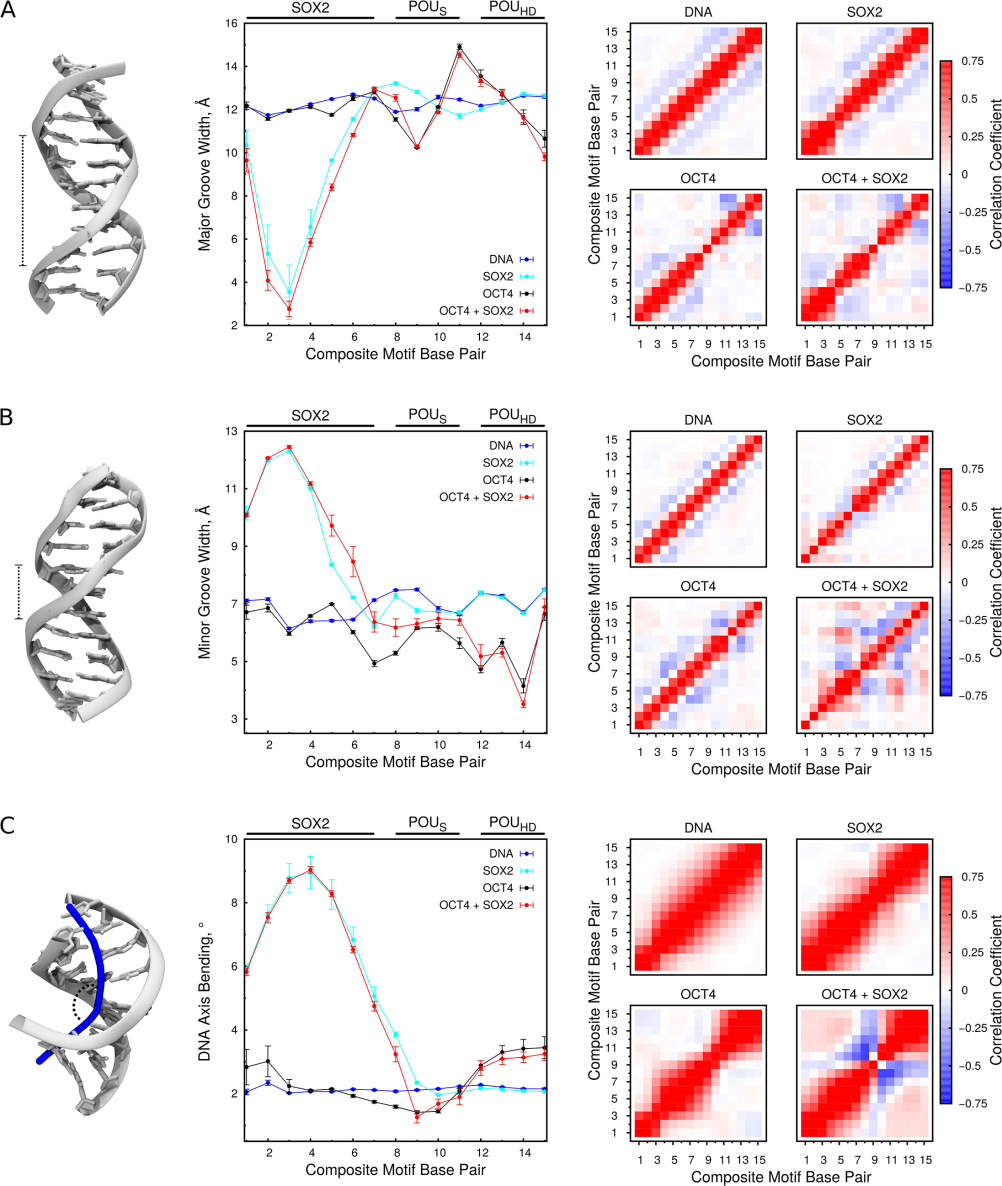
Effect of OCT4 and SOX2 binding on the DNA structural properties. (A) Major groove width (B) Minor groove width (C) Axis bending. On the left schematic representations of analyzed properties are drawn. The plots in the middle show the average values with standard errors (see Methods), whereas the plots on the right the inter base pair correlations. The data was collected from the four ensembles of unbiased simulations (free DNA, and SOX2-DNA, OCT4-DNA, OCT4-SOX2-DNA complexes).

To explore the DNA structural changes induced upon binding of OCT4 and SOX2 and how these may contribute to cooperativity and DNA-mediated allostery, we performed two additional ensembles of 1.5 μs unbiased simulations each, one for the SOX2-*UTF1* complex and one for the free *UTF1* DNA. Each ensemble was composed of 2 independent, 750-ns-long simulations. Then, we analyzed changes in the major (Fig 4A) and minor (Fig 4B) groove widths and axis bending (Fig 4C) in all the simulations relative to the free DNA.

SOX2 modifies the structure of the DNA by binding to the minor groove and inserting a methionine side chain between two base pairs, which significantly bends the DNA [22] (Fig 4C). In the absence of OCT4, SOX2 widens the major groove, and changes the inter base pair correlation of the major groove width in the region of the POU_S_ binding site (Fig 4A). These changes may enhance the DNA-binding affinity of the POU_S_ in the presence of SOX2. In addition, SOX2 very slightly narrows the major groove at the end of the POU_S_ and beginning of the POU_HD_ binding sites, indicating that the binding of SOX2 propagates a signal through the DNA structure up to eight base pairs away from the most distorted base pair of its own binding site. Remarkably, the combined effect of OCT4 and SOX2 involves mainly changes in the inter base pair correlations of all DNA-structural properties analyzed (Fig 4). Importantly, only the ternary complex shows correlations between the SOX2 and POU_HD_ binding sites. While this effect could be POU_S_-mediated, it is most likely due to the DNA-mediated communication observed in the network analysis (Fig 3).

OCT4 also modifies the structure of the DNA, causing alternate narrowing and widening of the major groove (Fig 4A), and an overall narrowing of the minor groove (Fig 4B). This is consistent with the binding of its globular domains to the major groove and the preference of the highly positive N-terminal tail of homeodomains for narrow minor grooves due to the strong negative electrostatic potential found in these regions of DNA [25]. In addition, the POU_HD_ slightly bends the last bases of the POU binding site (Fig 4C). Notably, although the presence of SOX2 modifies the DNA-bound configuration of the POU_HD_, it does not affect the POU_HD_-induced changes in the structure of the composite motif. Similarly, it has been reported that other POU factors bend the DNA [26]. However, the isolated POU_HD_ did not bend DNA, and therefore the bending was attributed to the POU_S_ or the interaction between the two domains.

### SOX2 influences the unbinding profiles of both OCT4 domains

To characterize the unbinding process of OCT4, we performed umbrella sampling simulations to dissociate each domain of OCT4 from the DNA in the absence and presence of SOX2. For this, we used the minimal interatomic distance between the pulled domain and the DNA (*d*_min_) as collective variable to describe the dissociation process. We only simulated the unbinding process when the other domain remained bound to the DNA because simulations with both domains detached are unlikely to converge on a reasonable timescale. To monitor the unbinding process we calculated how the recurrent and the non-stable (formed in less than 50% of the simulation time) interactions break during the simulations.

**Fig 5:**
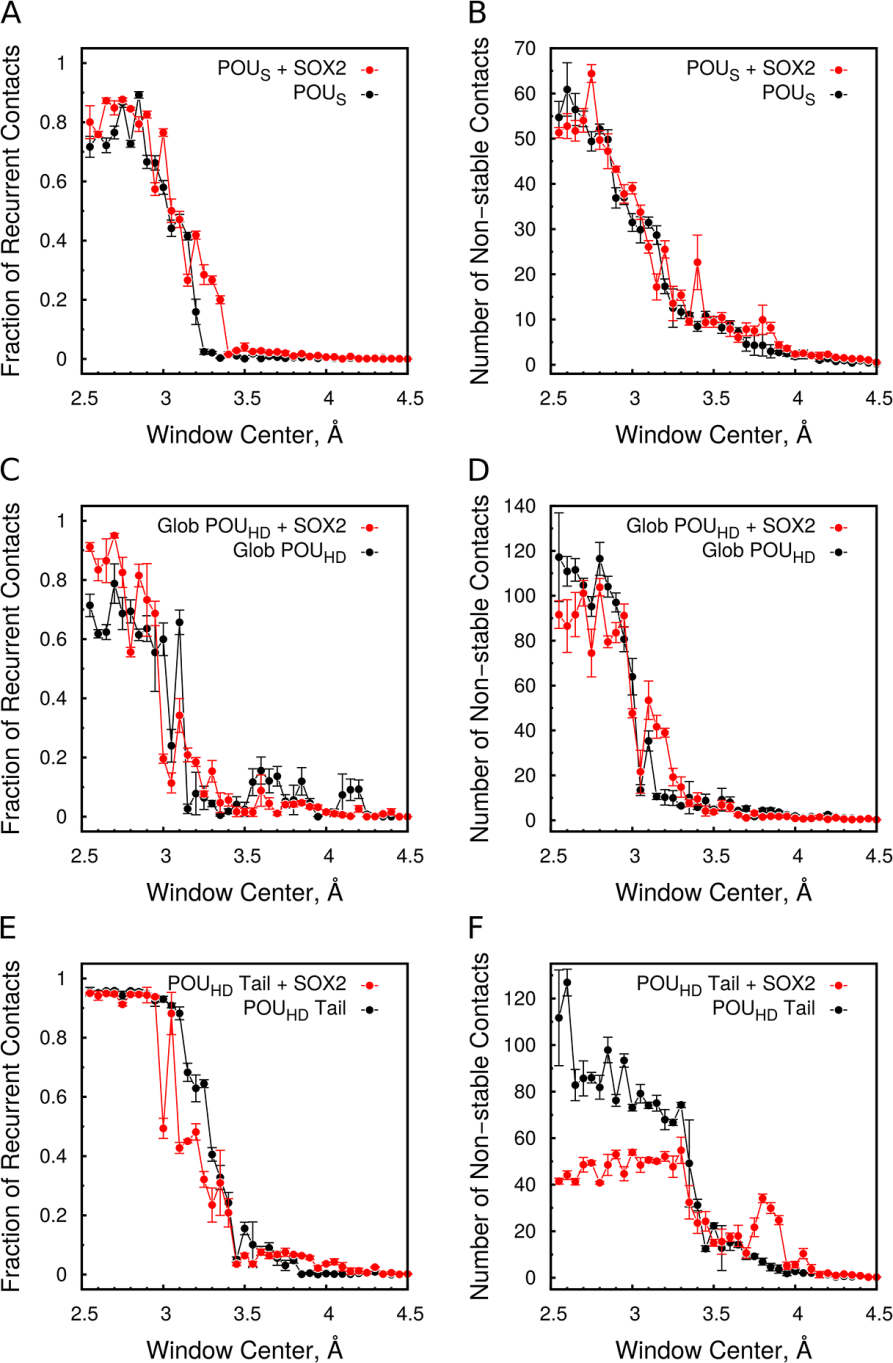
Unbinding profiles of the OCT4 domains. The change in the number of protein-DNA contacts is shown during the unbinding simulations while pulling the POU_S_ (A,B) or the POU_HD_ (C-F). (C,D) Globular region of the POU_HD_. (E,F) N-terminal tail of the POU_HD_. The contacts were divided into recurrent (A,C,E) and non-stable (B,D,F). The fraction of recurrent contacts was calculated using the number of recurrent contacts from the unbiased simulations as reference.

Irrespective of the domain analyzed or the presence of SOX2, most of the contacts were lost between 3.1 and 3.5 Å minimal distance separation (Fig 5). This suggests that the OCT4-*UTF1* interface is dominated by hydrogen bond interactions. The POU_S_ domain detaches from the DNA in a cooperative fashion, where all the recurrent interactions break simultaneously in the region with *d*_min_ between 3.0 and 3.2 Å (Fig 5A). The presence of SOX2 moves the upper limit of this region further to 3.4 Å (Fig 5A). On the other hand, SOX2 has no effect on the non-stable contacts between the POU_S_ and the DNA (Fig 5B).

Similar to the unbinding of the POU_S_, the unbinding of the globular region of the POU_HD_ is very cooperative, with all interactions breaking simultaneously (Fig 5C and 5D). Most of the recurrent contacts between the POU_HD_ tail and the DNA break in the region between 3.0 and 3.45 Å when SOX2 is absent (Fig 5E). Interestingly, the presence of SOX2 slightly decreases the lower limit of this region to 2.9 Å. In addition, there is an increase in the number of non-stable contacts in the region between 3.5 and 4.0 Å (Fig 5F). This shows that the protein-DNA interactions formed by the tail break at a larger separation than those formed by the globular region. The difference in the unbinding process of the POU_HD_ tail and the globular region is in agreement with our observation that these regions are independent in the correlation and communities analysis of the unbiased simulations (Fig 3A, 3B, and S4).

**Fig 6:**
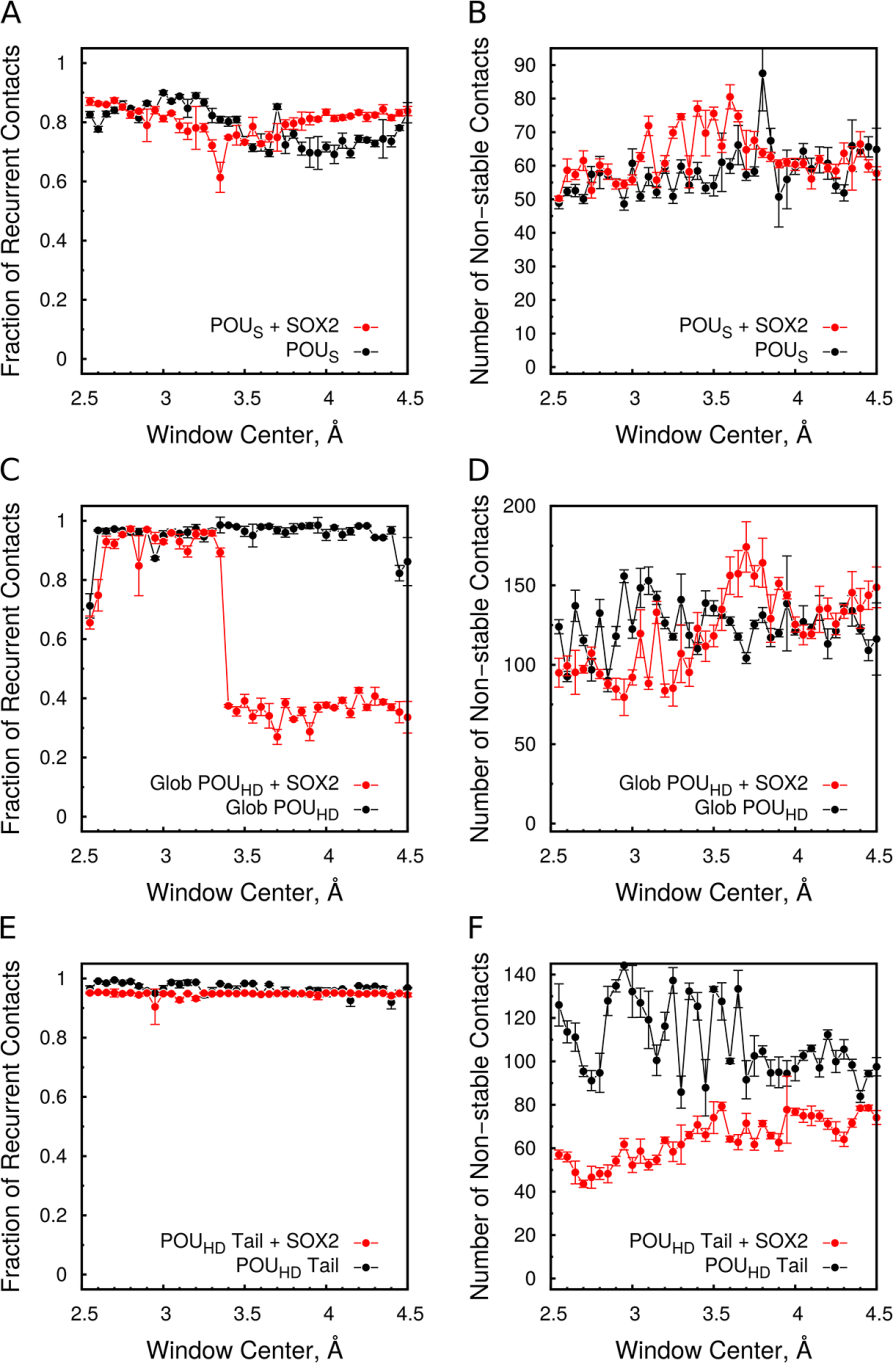
Interactions with the DNA of the remained-bound domain. (A,B) Change in recurrent (A) and non-stable (B) POU_S_-DNA contacts when pulling the POU_HD_. (C-F) Change in recurrent (C,E) and non-stable (D,F) contacts between the globular region (C,D) and the N-terminal tail (E,F) of the POU_HD_ when pulling the POU_S_. The fraction of recurrent contacts defined as in Fig 4. See also S5 Fig.

Next, we analyzed the effect of the unbinding of one domain of OCT4 on the domain that remained bound to DNA (Fig 6). The unbinding of the POU_HD_ has no impact on the number of recurrent or non-stable contacts of the POU_S_ (Fig 6A and 6B). On the other hand, the unbinding of the POU_S_ has a strong effect on the DNA interaction of the globular region of the POU_HD_ when SOX2 is present (Fig 6C and 6D). A significant decrease in the number of recurrent interactions is accompanied by an increase in non-stable interactions. This suggests that the POU_HD_ is reorienting, but not detaching from the DNA. Indeed, the analysis of the Rock and Tumble angles during dissociation further confirms this (S5 Fig). Importantly, the absence of this phenomenon in the simulation of the OCT4-*UTF1* complex suggests that this reorientation is induced by the allosteric communication between the POU_HD_ and SOX2. Conversely, the unbinding of the POU_S_ does not affect the number of recurrent and non-stable contacts between the POU_HD_ tail and the DNA (Fig 6E and 6F).

### Unbinding of either domain of OCT4 induces distal changes in DNA structure

To study the DNA structural changes upon unbinding of the OCT4 domains, we monitored the relaxation of the minor groove width and the bending angle for each base pair along the unbinding simulations.

**Fig 7:**
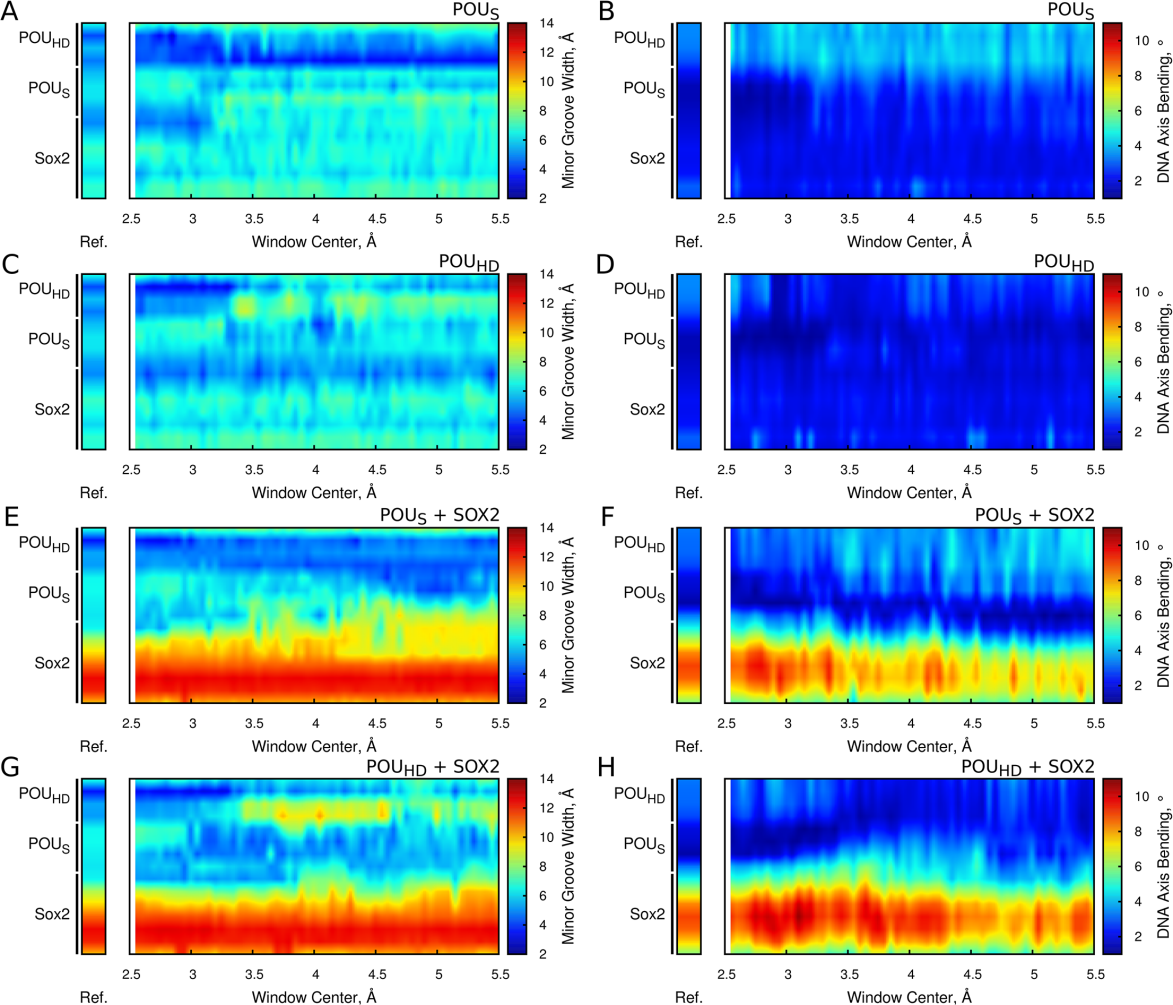
Changes in DNA structure during the biased simulations. The effect of the unbinding on the conformation of the composite DNA motif is shown in the absence (A-D) or the presence (E-H) of SOX2. The pulled domain was the POU_S_ (A,B,E,F) or the POU_HD_ (C,D,G.H). The properties analyzed were the minor groove width (A,C,E,G) and the bending of the DNA axis (B,D,F,H). For each plot, the average obtained from the unbiased simulations is shown as a color bar reference.

In the absence of SOX2, the unbinding of the POU_S_ produces a small widening of the minor groove in the region between the SOX and POU_S_ binding regions (Fig 7A) without affecting the bending angle (Fig 7B). Conversely, the unbinding of the POU_HD_ has a strong effect on the DNA structure. Around a minimal distance of 3.25 Å, the minor groove width at the 3' region of the POU_HD_ binding site increases towards the average value from ideal B-DNA (~ 5.4 Å) (Fig 7C), while the POU_HD_-induced bending of the DNA disappears as the POU_HD_ unbinds (Fig 7D). In addition, in the region between 3.5 and 4.5 Å, the detachment of the POU_HD_ tail from the DNA widens the minor groove at the beginning of the POU_HD_ binding site (Fig 7C).

Strikingly, when SOX2 is present, the changes in DNA structure induced by unbinding of both domains of OCT4 are affected. For instance, the POU_S_-induced narrowing of the minor groove seen in the absence of SOX2 (Fig 7E) is no longer present. Instead, when the POU_S_ unbinds from the DNA, the minor groove deformations induced by the POU_HD_ tail and SOX2 propagate into the POU_S_ binding region (Fig 7E). In addition, there is an increase in the POU_HD_ bending angle (Fig 7F), suggesting that the POU_S_ modulates the changes in DNA structure induced by SOX2 and the POU_HD_. The presence of SOX2 also significantly amplifies the widening of the minor groove induced by the unbinding of the POU_HD_ tail (Fig 7G), in agreement with the increase of non-stable contacts observed between this region and the DNA (Fig 5F). Remarkably, the unbinding of either domain from OCT4 causes a decrease in the bending angle induced by SOX2 (Fig 7F and 7H). Although this effect is stronger during the unbinding of the POU_S_ (Fig 7F), it is also present when the POU_HD_ dissociates (Fig 7H). Therefore, the changes in DNA structure induced upon unbinding of the POU_S_ and POU_HD_ propagate away from their binding regions, further demonstrating that the communication between the POU_S_-SOX2 interface and the POU_HD_ is DNA-mediated.

### SOX2 affects the relative DNA-binding strength of the OCT4 domains

To quantify the effect of SOX2 on the DNA-binding affinity of OCT4, we calculated the unbinding free energy profile for each of its domains from the biased simulations. Then, we calculated a macroscopic binding free energy from the total probability of finding the domains in the bound or unbound configurations (see Methods and S1 Text).

Because an experimentally measured affinity of OCT4 for this enhancer is not available, a direct comparison with the absolute affinities calculated here is not possible. However, an OCT4-SOX2 cooperativity of -1.56 kcal/mol has been measured for an idealized canonical motif [14,24], similar to the OCT1-SOX2 cooperativity of -1.7 kcal/mol measured for the enhancer of *HOXB1* [27]. Assuming that the presence of SOX2 neither modifies the cooperativity between the POU_S_ and the POU_HD_, nor the effect of the linker between them, we can calculate the OCT4-SOX2 cooperativity from our simulations as the sum of the SOX2-induced changes in the affinity of both domains (See S1 Text). For this, we tested several definitions of the bound-unbound threshold (Table 1).

**Table 1:** Estimates of the DNA-binding affinity of the POU_S_ and the POU_HD_.

Bound/Unbound Threshold	Without SOX2		With SOX2		OCT4-SOX2 Cooperativity
	POU_S_	POU_HD_	POU_S_	POU_HD_	
3.1 Å	-14.05 ± 0.05	-12.94 ± 0.12	-13.40 ± 0.10	-12.63 ± 0.11	0.96 ± 0.20
**3.3** Å	**-14.34** ± **0.05**	**-16.14** ± **0.08**	**-16.72** ± **0.07**	**-15.76** ± **0.09**	**-2.00** ± **0.15**
**3.5** Å	**-14.51** ± **0.05**	**-16.74** ± **0.07**	**-17.25** ± **0.07**	**-16.59** ± **0.11**	**-2.59** ± **0.16**
3.7 Å	-14.69 ± 0.05	-17.13 ± 0.05	-17.71 ± 0.06	-17.60 ± 0.13	-3.49 ± 0.16
3.9 Å	-14.87 ± 0.05	-17.32 ± 0.05	-18.01 ± 0.06	-18.69 ± 0.07	-4.51 ± 0.12
4.1 Å	-14.06 ± 0.05	-17.47 ± 0.05	-18.21 ± 0.06	-19.05 ± 0.07	-4.73 ± 0.12
4.3 Å	-14.29 ± 0.05	-17.59 ± 0.05	-18.38 ± 0.06	-19.29 ± 0.07	-4.79 ± 0.12

All values are in kcal/mol. The two values for the unbound/bound threshold that are most consistent with experiment and reflect the breaking of most recurrent contacts during the biased simulations.

**Fig 8:**
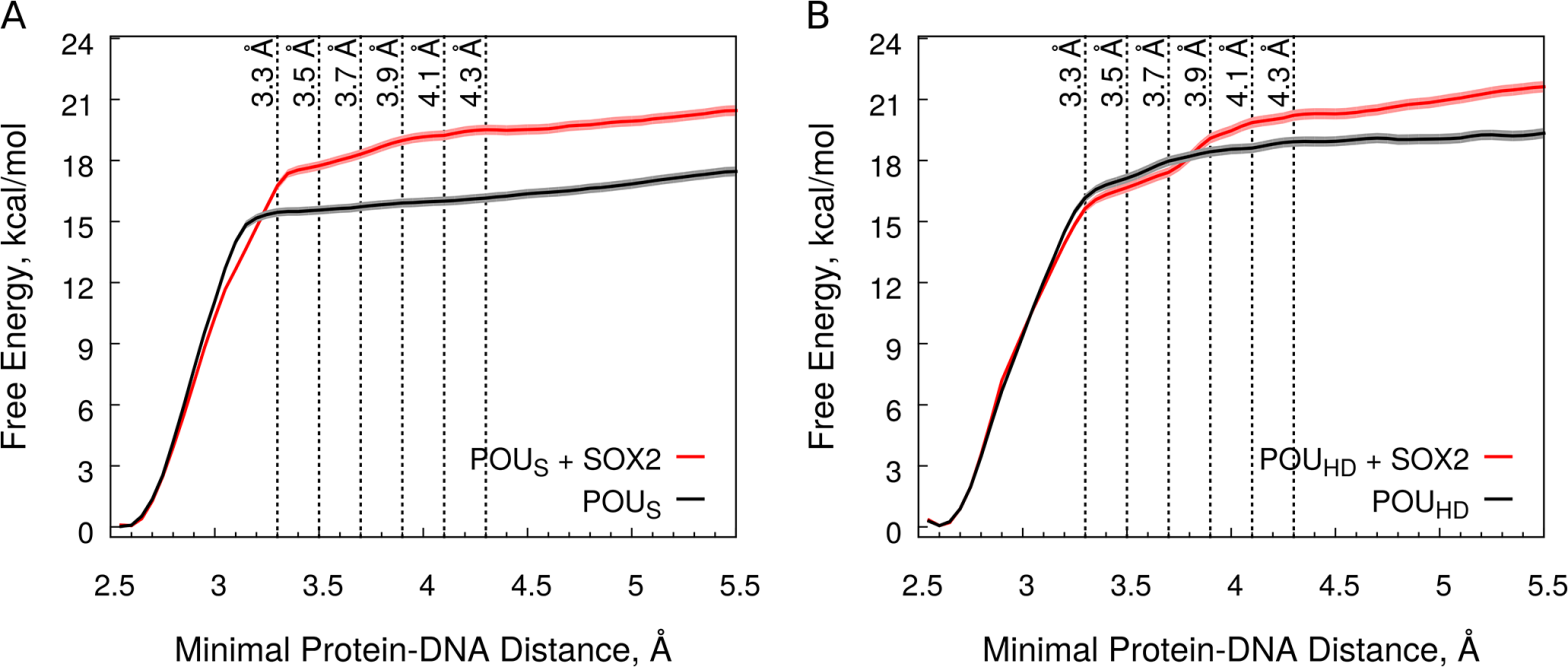
Unbinding free energy profiles. (A) POU_S_. (B) POU_HD_. The black and red curves show the profiles in absence and presence of SOX2 respectively. The dotted vertical lines mark the different bound-unbound thresholds tested (Table 1.). The shaded lines represent the error calculated as described in Methods. See also S6 Fig.

As all our free energy profiles show a sharp transition close to the hydrogen bond distance threshold (3.2–3.4 Å) (Fig 8), which reflects the breaking of most recurrent contacts around the same values (Fig 5), we consider that the bound-unbound transition is well defined at thresholds between 3.3 and 3.5 Å. Using these values, we calculated cooperativities of -2.0 and -2.59 kcal/mol respectively (Table 1), in good agreement with experiments [14,24,27,28]. Lower values for the threshold are not appropriate as they correspond to the steep part of the free energy profiles, whereas higher values still provide the correct sign for the cooperativity although they overestimate it, possibly due to the poor sampling of the unbound state (Table 1, S6 Fig). Therefore, we focus on the results obtained with threshold values of 3.3 and 3.5 Å. We stress that the comparison with experiments is only approximate, as neither the DNA-binding element nor the ionic strength were the same.

In the absence of SOX2, the POU_HD_ binds to DNA stronger than the POU_S_ by 1.8–2.2 kcal/mol despite the reduced number of sequence-specific POU_HD_-DNA interactions (Table 1, Fig 8). This is consistent with the affinities measured for the isolated domains of OCT1, where the POU_HD_ binds 2.9 kcal/mol stronger than the POU_S_ to a DNA with consensus sequence [29]. Although the difference between our estimation and the experimental value in the case of OCT1 may be attributed to differences between the two proteins, it may also reflect the absence of sequence-specific POU_HD_-DNA interactions in the OCT4-*UTF1* complex. The presence of SOX2 results in an increase in the unbinding free energy profile of the POU_S_ (Fig 8A), corresponding to an increase in DNA-binding affinity of 2.4–2.7 kcal/mol (Table 1). This agrees with the shift towards a higher minimal distance for the breaking of recurrent POU_S_-DNA contacts in the presence of SOX2 (Fig 5A). Importantly, SOX2 also modifies the unbinding free energy profile of the POU_HD_ (Fig 8B). This involves a small decrease in the region between 3.2 and 3.75 Å of the free energy profile combined with an increase in the region between 3.75 and 4.0 Å (Fig 8B). The first effect coincides with the shift in the breaking of the recurrent POU_HD_ tail-DNA contacts (Fig 5E), whereas the second correlates with the increase of non-stable POU_HD_ tail-DNA contacts (Fig 5F) and the widening of the DNA minor groove (Fig 7G). Overall, SOX2 decreases the DNA-binding strength of the POU_HD_ by 0.4 kcal/mol at an unbound/bound threshold of 3.3 Å or 0.15 kcal/mol at 3.5 Å (Table 1). Consequently, the DNA-binding strength of the POU_S_ becomes larger by 0.7–1.0 kcal/mol than that of the POU_HD_ in the presence of SOX2 (Table 1). Interestingly, the strong effect of SOX2 on the unbinding free energy profile of POU_S_ corresponds to a modest effect on its orientation and dynamics, whereas a large effect on the POU_HD_ dynamics corresponds to a smaller effect on its unbinding free energy profile (Figs 2 and 8). Remarkably, the increase in the DNA-binding affinity of the POU_S_ is larger than the measured OCT4-SOX2 cooperativity. The estimated cooperative binding free energy approaches the experimental value only with the additional decrease in the POU_HD_ affinity (Table 1), which further supports the finding of an allosteric component that modulates the OCT4-SOX2 cooperativity and suggests that the allostery has a slight detrimental effect on the cooperativity.

Our estimates of the DNA-binding affinity are higher than expected for typical transcription factors, and thus unlikely to represent the real values. In principle, if we account for the inter-domain cooperativity and the effect of the linker peptide, we can compare our estimations with the previously measured affinities of the POU_S_ and POU_HD_ domains of OCT1 which are -7.9 and -10.8 kcal/mol respectively [29]. These values only account for the inter-domain cooperativity. Although we cannot compare directly the effects of the linker peptides of OCT4 and OCT1 due to their different length and structure [20] (See S1 Text), the much lower affinities measured for the isolated domains of OCT1 indicate that we overestimate the absolute binding free energies

The analysis of the consistency in the density of states shows that we achieved convergence at minimal distances lower than 4 Å, but the quality of the sampling decreases at high minimal distances (S6 Fig). This is expected, since the volume of the conformational space increases significantly with the protein-DNA separation distance and is therefore a common problem in this type of calculations [30]. A confinement scheme in which conformational, positional and rotational restraints are used and removed after the induced dissociation has successfully been used to alleviate such issues in protein-ligand systems [30,31,32]. However, the very high degeneracy of RMSD-based conformational restraints makes this approach unlikely to converge in our case, for which both partners are very large and flexible. The imperfect convergence at higher separation as well as the presence of the second domain bound to the DNA may explain the inaccuracy in the estimation of binding free energies. However, our estimates for the OCT4-SOX2 cooperativity as well for the difference in the affinities of the POU_S_ and POU_HD_ in the absence of SOX2 are in good agreement with experiment, suggesting that the error in the calculation is of similar magnitude among the different free energy profiles.

## Discussion

Our simulations demonstrate that the mechanism by which OCT4 recognizes the DNA is modified by SOX2. Interestingly, a similar phenomenon has been described for OCT1. NMR studies have shown that the POU_S_ of OCT1 is involved in hopping between DNA segments, while the POU_HD_ scans the DNA through 1D sliding. Interestingly, co-binding with SOX2 to the *HOXB1* enhancer increases the affinity of the POU_S_ locking it on its cognate site [27,33]. This has also been inferred from steered molecular dynamics of the complex between SOX2 and the POU_S_ from OCT1 [34]. As a result, the POU_HD_ is now the most likely to transfer to another region of the DNA through a “slide and transfer” mechanism [33]. From our simulations, we observed that SOX2 also locks the POU_S_ of OCT4 on its binding site, as its DNA binding affinity increases by 2.4–2.7 kcal/mol (Table 1). In addition, we show that the presence of SOX2 generates an allosteric signal that propagates through DNA, couples the POU_HD_ motions with the major groove of the DNA (Fig 2) and likely decreases the POU_HD_ affinity beyond that of the POU_S_ (Table 1). This suggests that the POU_HD_ of OCT4 also adopts the exploratory role when SOX2 is present. Therefore, our findings suggest that, by modulating the affinity and specificity of the individual domains, SOX2 can affect the mechanism by which many POU factors recognize the DNA. However, a mechanism involving DNA-mediated allostery has not been yet described for any other POU-SOX2 complex. Further research is necessary to understand if such a mechanism is common among POU factors. Nevertheless, the POU-SOX2 cooperativity is likely to change the identity of their target genes, and the transcriptional programs they promote.

For OCT4, this regulatory mechanism may have important functional implications. A ChIP-based analysis has suggested that OCT4 does not bind in combination with SOX2 during the early stages of reprogramming to pluripotency [19]. Remarkably, the analysis of the binding sites shows that most of the DNA specificity comes from the POU_HD_. On the other hand, the same analysis in pluripotent cells shows that the POU_S_ has a much stronger specificity than the POU_HD_ [13]. Therefore, the selection among different DNA recognition mechanisms induced by SOX2 may give cellular and genomic context specificity to the biological function of OCT4.

Our simulations show that the unbinding of the POU_S_ involves the breaking of all the protein-DNA contacts simultaneously (Fig 5A and 5B). On the other hand, the POU_HD_ shows a modular behavior where the domain can be further sub-divided into the N-terminal tail and the globular region. When binding to a consensus sequences, the globular region is the one that contributes most to the DNA specificity, because its docking helix forms sequence-specific contacts with DNA bases. However, this is likely to be a small contribution to the total DNA-binding affinity given that estimates of the affinity of some homeodomains for unspecific sequences have shown that they can strongly bind the DNA in the absence of their cognate site (K_d_ ~ 300 nM) [35]. Notably, the interaction between the N-terminal tail and the minor groove seems to determine part of the DNA-binding specificity of homeodomains [25], since different tail sequences prefer DNA sequences with different minor groove widths. In turn, this correlates with the sequence preferred by the globular region of the homeodomain. Furthermore, protein-protein interactions involving the N-terminal tail of homeodomains are known to drastically change their sequence preferences, thus evoking latent specificities [3]. Theoretical and experimental studies have shown that the tail of homeodomains has a major contribution to the overall binding affinity and is key for the DNA-recognition mechanism, as it can speed up the binding process [36,37]. We showed that SOX2 modifies the dynamics of the POU_HD_ by enhancing its interaction with the DNA backbone through an allosteric signal that propagates through its N-terminal tail (Fig 3C). It is possible that this changes the sequence selectivity of OCT4, thus promoting the binding to degenerated binding sites such as the *UTF1* sequence. Similarly, the presence of SOX2 allows the OCT4 homolog OCT1 to bind a composite motif where the POU_HD_ half-site has been removed [38]. In addition, our findings suggest that this region contributes the most to the affinity of the POU_HD_. Interestingly, the POU_HD_ tail serves as the nuclear localization signal of OCT4 [39], and the region around it is subject to several post-translational modifications known to alter its biological activity [10]. Therefore, the allosteric communication is likely to be a key aspect of the regulation of OCT4's function *in vivo*.

A key difference between OCT4 and most POU factors is the presence of a significantly more structured linker peptide that contains a defined helix (α_5_). Previously, we have demonstrated that the structure of this region is important for protein-protein interactions [20,24]. Thus, our initial hypothesis was that this region serves as a communication route between the two domains of OCT4 and between OCT4 and SOX2 in ternary complexes. However, the network analysis shows that the communication occurs mainly through the DNA and not the protein (Figs 3C and 3D and S4). This suggests that the allosteric communication pathway is not OCT4-specific and may represent a general mode of communication between POU and SOX proteins.

Furthermore, the unbinding of the domains reveals subtle modifications of the DNA structure that propagate to the adjacent biding sites, consistent with a DNA-mediated allosteric signal that is likely to modify protein-DNA binding affinities. Interestingly, a similar effect has been described for other assemblies of transcription factors. For instance, the expression of interferon-β depends on the cooperative assembly of 8 proteins to a 55 bp binding site [5]. Structural studies have suggested that their cooperativity arise from DNA-mediated effect, as their protein-protein interfaces are very small and flexible [7,40]. In addition, allosteric effects through the DNA have been systematically explored using synthetic binding sites, and they have been shown to be key to the binding and activity of other transcription factors [4,6]. In one case, it has been observed that an interplay between direct and allosteric interactions is key for the assembly of protein-protein-DNA complexes [41]. However, allostery accounted for a positive contribution to DNA-binding cooperativity and has not been related to the selection of alternative DNA-recognition mechanisms.

In the case of the OCT4-SOX2 interaction upon binding to the *UTF1* enhancer, the allosteric component may have a small negative contribution to the cooperativity. Therefore, DNA-mediated allostery can determine the cooperativity of proteins that bind to DNA not close enough to form physical interactions or modulate cooperativity in an interplay with protein-protein interactions. The latter provides a mechanism to modify DNA exploration pathways, which in turn may give an additional layer of specificity to enhanceosome assembly and transcriptional regulation.

## Methods

### Molecular dynamics simulations

We built models of the OCT4-SOX2-*UTF1* ternary complex with the human *UTF1* sequence (5'-CAGGCATTGTTATGCTAGCGGAACTCC-3') using our previous models of OCT4-SOX2 complexes bound to a consensus canonical motif (*HOXB1* enhancer) [20]. These were built based on the structure of the OCT1-SOX2-*HOXB1* complex (pdbid 1O4X) [15] and the OCT4-OCT4-DNA homodimer complex [20] (see S1 Text for details). Two alternative models of the OCT4-SOX2-*UTF1* complex, identical in the DNA-binding interface but slightly differing in the unstructured part of the linker region, were chosen to provide slightly different strating coordinates. These and the corresponding models of the OCT4-*UTF1* complex were equilibrated in explicit TIP3P water and 150 mM NaCl under periodic boundary conditions (see S1 Text for details). Typically, the systems contained ~ 100.000 atoms. Four independent, 450 ns-long simulations of the ternary complex and the corresponding simulations of the binary complex were performed in the canonical (NPT) ensemble. Two simulations were performed for each model, each of them with different initial velocities, using a standard protocol in NAMD [42] (details in S1 Text).

To generate the starting configuration for the simulations of the SOX2-*UTF1* complex and the free *UTF1* DNA, OCT4 and 13 Cl^-^ ions compensating for the OCT4 net charge of +13 were stripped from the starting configurations of the solvated OCT4-SOX2-*UTF1* and OCT4-*UTF1* systems respectively. The newly generated SOX2-*UTF1* and *UTF1* systems were equilibrated (see S1 Text for details) and two independent 750-ns-long simulations, with different initial velocities, were performed using the same protocol as described above.

For proteins and DNA we used the amber force field [43] modified for DNA (ff99) [44] and proteins (ff99SB) [45] with further corrections for protein side-chains (ILDN) [46], protein backbone (NMR-based) [47] and DNA backbone [48]. For the ions we used the Smith-Dang parameters [49]. The integration step was 1.5 fs and coordinates were saved every 3 ps.

### Unbinding free energy profiles

We performed umbrella sampling simulations to calculate the free energy profiles for the unbinding of the OCT4 domains from the DNA. Most of the errors in unbinding free energy profiles come from the incomplete sampling of the unbound state [30]. In the case of multi-domain proteins such as OCT4, obtaining converged sampling of the state in which all domains are dissociated from the DNA is particularly challenging. Therefore, we only estimated the free energy profiles for the dissociation of one domain, while the other remained bound. We defined the POU_HD_ as residues 95 to 152 and the POU_S_ domain as residues 1 to 88, which includes the helical region of the linker (helix α_5_; residues 76 to 88) (Fig 1A). The collective variable chosen to describe the dissociation process was the minimal interatomic distance between the pulled domain and the DNA (*d*_min_) [50]. In each window, *d*_min_ was restrained by a biasing potential of the form

$$U\left(d_{\text{ij}}\right)=\{\begin{array}{l} \sum k{\left(d_{\text{ij}}-d_{\text{min}}^{c}\right)}^{2},{\text{if d}}_{\text{min}}<{\text{d}}_{\text{min}}^{c}\\ \;k{\left(d_{\text{ij}}-d_{\text{min}}^{c}\right)}^{2},{\text{if d}}_{\text{min}}⩾{\text{d}}_{\text{min}}^{c} \end{array}$$

where $d_{\text{min}}^{c}$ is *d*_min_ at the center of the window. When at least one protein-DNA heavy atom pair has a distance shorter than $d_{\text{min}}^{c}$ then all atom-atom pairs ij with distances *d*_ij_ below this threshold are subjected to the biasing potential. Otherwise, only the pair ij with the minimal distance is biased. This definition of the potential limits the bias imposed on the dissociation mechanism. While no atom pair can be closer than the current value of *d*_min_, atom pairs can potentially be further apart, which does allow for progressive, hierarchical unbinding. This is a reasonable assumption considering that short-range Van der Waals contacts typically break before stronger long-range electrostatic interactions. Indeed, such hierarchical behavior has been seen in previous studies on other protein-DNA [50] and protein-protein [51] systems using the same enhanced sampling methodology.

Each domain was pulled away from the DNA in the presence and absence of SOX2. We started the first window from a configuration taken after 57.8 ns of unbiased simulation. For each free energy profile, we used 60 windows centered every 0.05 Å, covering a range for *d*_min_ between 2.55 and 5.5 Å.

To minimize the equilibration time, each biased simulation was started after 4.5 ns of simulations of the previous window. For each window we performed 22.5 ns of biased simulation, summing up to 1.35 μs simulation time per domain, per system. We used a force constant of 300 kcal /mol ∙ Å^2^ for most windows. For the unbinding of the POU_HD_ in the absence of SOX2, we used a force constant of 250 kcal /mol ∙ Å^2^ at separations above 4.75 Å, given that the free energy profile is essentially flat, and large biases are no longer necessary. For all further analysis, the initial 6 ns of simulation in each window were discarded as equilibration.

The free energy profiles were reconstructed from the umbrella sampling simulations using the weighted histogram method (WHAM) [52]. The error associated with each profile was calculated as described in [53]. The convergence of the simulations was assessed by comparing the density of states calculated for each pair of consecutive windows (See S1 Text and [53] for details).

A macroscopic binding free energy was calculated from the free energy profile, using the total probability of the domain being either bound or unbound. From this, the affinity can be calculated as,

$$\Delta \text{G}=-{\text{k}}_{B}T\text{ln}\frac{\int_{bound}^{}\rho \;\left(d_{\text{min}}\right){\text{dd}}_{\text{min}}}{\int_{unbound}^{}\rho \;\left(d_{\text{min}}\right){\text{dd}}_{\text{min}}}$$

where *ρ*(*d*_min_) can be estimated from the relation *ρ*(*d*_min_) ∝ exp(*G*(*d*_min_)/*k*_*B*_ *T*).

Given the absence of a clear transition state in the free energy profiles, we defined the boundary between the bound and unbound states at different values of *d*_min_. As an upper integration limit we always used 5.5 Å. Importantly, due to the exponential nature of the integration, the lower region of the unbound state contributes the most to the final result, and the choice of the upper integration limit does not change the results significantly. However, the definition of the integration limits is arbitrary and can modify the final result [54]. The errors on the computed binding free energies were obtained from the errors on the PMF by applying linear error propagation theory to the equation used to calculate ΔG (see above), as implemented in the ‘Uncertainites’ Python package (http://pythonhosted.org/uncertainties).

### Analysis of structural properties

The structural properties of the DNA were analyzed using Curves+ [55]. The standard errors were generated using the block averaging procedure [56].

All other properties were analyzed using VMD [57]. To measure the orientation of the binding domains on the DNA, we created a DNA-based coordinate system as described previously [24]. We defined v_x_ as the vector between the centers of mass of the first and last base pairs of the OCT4 binding site, v_t_ as the vector between the backbone of the bases from the first base pair of the OCT4 binding site, v_z_ as the cross product of v_x_ and v_t_, and v_y_ as the cross product of v_x_ and v_z_. The “Rock” angle is the angle between the axis of the docking helix of the domain analyzed and v_y_ projected on the v_y_/v_z_ plane. The “Tumble” angle is the angle between the axis of the docking helix of the domain analyzed and v_x_ projected on the v_x_/v_z_ plane (Fig 2A and 2B). The conformational volume sampled inside the two-dimensional rock/tumble subspace was estimated from a principal component analysis of the rock/tumble data sets for the POU_S_ for which the corresponding population density is quasiharmonic. The ratio of conformational volumes sampled in the absence and presence of SOX2 was computed as the ratio of the areas of the corresponding confidence ellipses, which only depends on the product of the square roots of the covariance matrix eigenvalues if the same (arbitrary) confidence threshold is chosen in both cases.

The average and standard errors for the structural properties of the biased simulations were calculated without re-weighting. Although this influences the absolute values of the properties, it will not affect the comparative analysis.

### Network analysis

We performed a contact network analysis with the VMD network plugin [58] which uses Carma [59] to calculate positional cross-correlations. For this, we defined nodes on the C_α_ and C_β_ of the proteins. For prolines, glycines, and alanine only one node on the C_α_ was defined. For DNA we defined two nodes per nucleotide, one representing the backbone centered on the C3' atom and one representing the base centered on N3 in adenines and guanines, or C4 in cytosines and thymines. This selection of nodes is representative of the description of the dynamics of the backbone, protein side-chains and DNA bases and represents both the major and minor groove faces of DNA. To define the edges of the network, an atom-atom contact map was calculated where only those contacts present in more than 75% of the simulation time were kept. A contact was defined as a pair of atoms separated by 4.5 Å or less. Then, an edge was added between two nodes when at least 1 atom-atom contact exists between the atoms represented by the beads. Edges were not allowed within the same protein residue and between neighboring protein and DNA backbone beads. To explore possible communication pathways between the POU_HD_ and SOX2, we calculated the shortest collection of paths between the POU_S_-SOX2 and POU_HD_-DNA interfaces. Here, the inter-nodal distance *d*_mn_ is defined as *d*_mn_ = -log|*C*_mn_|, where *C*_mn_ is the positional cross-correlation coefficient between two nodes of the network. Then, we included only paths with inter-nodal distances within 5 of the optimal path.

## Supporting Information

S1 Text Supporting document with detailed methods. References are cited with their numbers either from the main article or from the additional list at the end of this document which contains those not cited in the main article.
(PDF)

S1 Table Number of protein-DNA stable contacts seen in the unbiased simulations of the OCT4-*UTF1* and OCT4-SOX2-*UTF1* systems (DOC)

S2 Table Principal component analysis of the Rock-Tumble data sets for the POU_S_ domain (DOC)

S1 Fig OCT4-DNA contacts during the unbiased simulations. (A) Average number of OCT4-DNA contacts per-residue. The graph on top shows the number of protein-DNA contacts present in a model of the OCT4-SOX2-*HOXB1* complex. The gray boxes highlight the 8 helices of OCT4. α_1_ – α_4_ correspond to the POU_S_, while α_6 –_ α_8_ to the POU_HD_. (B,C) Evolution of the number of DNA contacts made by the globular region of the POU_HD_ in the absence (B) and presence (C) of SOX2 during the six independent 450 ns-long unbiased simulations. See also Fig 1.
(TIF)

S2 Fig Recurrent OCT4-DNA and the OCT4-SOX2 interactions during the unbiased simulations. (A,B) Recurrent protein-DNA interactions of the POU_S_ and POU_HD_ in the absence (A) and presence (B middle, right) of SOX2. (B) Protein-protein recurrent contacts at the OCT4-SOX2 interface (left). (C,D) SOX2-induced changes in recurrent protein-DNA interactions with the DNA bases (C) or backbone (D) mapped on the structure of the OCT4-SOX2-*UTF1* complex. The color scale shows the difference in recurrent contacts, measured as Q_+SOX2_—Q_−SOX2_. See also Fig 1.
(TIF)

S3 Fig Contribution of each simulation to the overall orientational dynamics in the DNA-bound configurations. Rock-Tumble measurements in the absence (C,D) or presence (E,F) of SOX2 for the POU_S_ (C,E) and POU_HD_ (D,F). The black lines represent the histograms shown in Fig 2. and are located at 4, 40, 400, 4000 counts. See also Fig 2.
(TIF)

S4 Fig Network analysis of correlated motions. The subnetwork communitites are shown in different colors. View (B) corresponds to view (A) rotated by 180° around the axis shown. See also Fig 3.
(TIF)

S5 Fig Effect of the unbinding of the domains of OCT4 on the orientation of the domain that remains bound to the DNA. Effect of the unbinding of the POU_HD_ on the orientation of the POU_S_ (A,B). Effect of the unbinding of the POU_S_ on the orientation of the POU_HD_ (C,D). The simulations were performed in the absence (A,C) or presence (B,D) of SOX2. The points show the average and the standard deviation of the Rock and Tumble values from each umbrella window. The color scale represents the protein-DNA separation of the domain being pulled. See also Fig 6.
(TIF)

S6 Fig Sampling inconsistency, θ_1,2_, between successive umbrella windows as a function of the inter-partner distance. (A,B) POU_S_ in the absence (A) or presence (B) of SOX2. (C,D) POU_HD_ in the absence (C) or presence (D) of SOX2. See also Fig 8.
(TIF)
