# Supplementary material for: Cooperative DNA Recognition Modulated by an Interplay between Protein-Protein Interactions and DNA-Mediated Allostery
Source: PLoS Comput Biol. 2015 Jun 11;11(6):e1004287. doi: 10.1371/journal.pcbi.1004287 (PMC4465831; doi:10.1371/journal.pcbi.1004287)
Supplement: S1 Text — References are cited with their numbers either from the main article or from the additional list at the end of this document which contains those not cited in the main article. (PDF) [file pcbi.1004287.s001.pdf]

Cooperative DNA recognition modulated by an interplay between protein-protein interactions and DNA-mediated allostery

Felipe Merino, Benjamin Bouvier, and Vlad Cojocaru

1 Modeling of the OCT4-*UTF1* and OCT4-SOX2-*UTF1* complexes

Previously, we created models of the OCT4-SOX2 complex bound to a consensus canonical motif (*HOXB1* enhancer) [20] based on the NMR structure of the OCT1-SOX2-*HOXB1* (PDBID 1O4X) [15] and the crystal structure of the OCT4 homo-dimer bound to a semi-palindromic site (PDBID 3L1P) [20]. From those structures, we modeled the OCT4-SOX2-*UTF1* complex by mutating the DNA bases from the *HOXB1* enhancer to reflect the *UTF1* sequence using SPDV [60] (5'-CAGGCATTGTTATGCTAGCGGAACTCC-3'). This is possible because both enhancers contain a composite motif with the same orientation and spacing between the individual binding sites. We further refined the models by optimizing the position of those side chains with no electron density in the crystal structure (residues 86 to 96) using a simulated annealing protocol described previously [24]. The OCT4-*UTF1* models were generated by removing SOX2 from the models of the ternary complexes. The orientation of glutamine, asparagine, and histidine residues and the position of structural waters was predicted using FoldX [61]. To avoid truncation artifacts, we methylated the 5' and 3' ends of DNA and the N-terminal of the proteins, and acetylated the C-terminal end of the proteins. Ionizable residues were assigned their standard protonation states at pH 7. The systems were solvated in a truncated octahedral box of TIP3P waters that extended at least 12 Å beyond any macromolecular atom. Then, the systems were neutralized with sodium ions and finally the ionic strength was adjusted by adding 150 mM NaCl. The final size of the systems was typically $\sim$ 100.000 atoms.

2 Equilibration protocol

For all simulated systems, we applied the following step-wise equilibration procedure. Initially, the protein and DNA heavy atoms were restrained to their starting positions using a harmonic restraint of 50 kcal/mol·Å^2. We did not apply this restraint to the first two and last three base-pairs or to the terminal amino acids of the proteins as these were incorporated after the optimization of the starting models. With these constraints, 2000 minimization steps were performed. Then, we performed 25 ps of isochoric simulations to slowly bring the systems to 298 K. The force constant for the restraint was further decreased to 10 kcal/mol·Å^2 and 250 ps of isobaric-isothermic (NPT) simulation were performed to adjust the pressure to 1 atm. After this, the restraints on residues 85 to 89 and the side chains of residues 90 to 97 of OCT4 (the linker region and the tail

of the POU_{HD}) were removed and 250 ps of simulation were performed. Later, the restrains on the DNA and all the remaining protein side chains were removed and another 250 ps of simulation were performed. Then, all positional restraints were removed. The helical conformation of the linker helix of OCT4 (residues 77 to 84) was restrained and 250 ps, followed by 500 ps of unrestrained simulations were performed. During all equilibration, the integration step was 1 fs. Finally, the integration step size was increased to 1.5 fs and 1.5 ns of unrestrained simulation were performed.

For the SOX2-DNA and the free DNA, the equilibration protocol was slightly modified because these systems do not involve multiple DNA binding domains, flexible linkers, or missing residues in the template structures used to build the models. The equilibration started with 25 ps isochoric simulation to bring the temperature to 298 K with all heavy atoms restrained. After this, two consecutive 250 ps of isobaric-isothermic simulation were performed to adjust the pressure to 1 atm, using positional restraints on all the heavy atoms of 10 and 1 kcal/mol·Å^2 respectively. Then, the restraints were kept only on the protein and DNA backbone and further 250 ps of NPT simulation was performed. Finally, the restraint was further lowered to 0.1 kcal/mol·Å^2 and a final 250 ps of restraint NPT simulations was performed. This was followed by 500 ps and 1 ns of unrestrained NPT simulations with the time step of 1 fs and 1.5 fs respectively.

3 Molecular dynamics simulations

All simulations were performed using NAMD [42]. The length of all bonds involving hydrogen atoms were constrained using the SHAKE [62] and SETTLE algorithms [63] for macromolecules and water respectively. The direct calculation of non-bonded interactions was performed using a cutoff of 10 Å. The long range electrostatic interactions were calculated using the particle-mesh Ewald algorithm [64]. To account for the truncation of the Lennard-Jones potential, a correction for the pressure and energy was applied [65]. The temperature was kept constant at 298 K with a Langevin thermostat (damping coefficient = 1 ps^{-1}), while the pressure was maintained at 1 atm using a Nose-Hoover-Langevin piston (Period = 200 fs, Decay = 100 fs). The two different models of the OCT4-SOX2-*UTF1* complex and the corresponding models of the OCT4-*UTF1* complex were equilibrated using the step-wise protocol described above.

4 Estimation of the binding cooperativity

The binding cooperativity between two transcription factors can be expressed as the energetic coupling of their binding affinities. Thus, the binding cooperativity of the OCT4-SOX2 combination can be written as

$$\Delta\Delta G_{\text{OCT4}}^{\text{SOX2}} = \Delta G_{\text{OCT4}}^{\text{SOX2}} - \Delta G_{\text{OCT4}} \tag{1}$$

where $\Delta G_{\text{OCT4}}^{\text{SOX2}}$ and ΔG_{OCT4} represent the affinity of OCT4 in the presence or absence of SOX2 respectively.

In principle, the DNA-binding affinity of a multi-domain transcription factor can be estimated from the

affinities of the isolated domains. For instance, let us consider a transcription factor with two DNA-binding domains (A and B) wich bind the DNA with dissociation constants K_A and K_B respectively. Upon the binding of the first domain A, the second domain will bind the DNA with a dissociation constant K_B/C_{Eff}, where C_{Eff} represents the effective concentration of the domain B due to the presence of the tethering linker [66]. Therefore, the affinity of the complete protein can be written as

$$K_{AB} = \frac{K_A K_B}{C_{\text{Eff}}} \tag{2}$$

In the case of the OCT1, experimental measures of the effect of the linker have shown that the effective concentration due to the tethering is $\sim$ 3.6 mM [29]. On the other hand, C_{Eff} has been calculated as 5.8 mM based only on a worm-like chain representation of the linker [66]. Unfortunately, this approximation is not necessarily valid in our case, due to the extra structure in OCT4's linker.

Nevertheless, assuming that the presence of SOX2 does not modify the effect of the linker, and given that $\Delta G = -k_B T \ln K$, we can combine equations 1 and 2 to calculate the OCT4-SOX2 cooperativity

$$\Delta\Delta G^{\text{SOX2}}_{\text{OCT4}} = \Delta\Delta G^{\text{SOX2}}_{\text{POU}_\text{S}} + \Delta\Delta G^{\text{SOX2}}_{\text{POU}_\text{HD}} \tag{3}$$

solely based on the effect of SOX2 on the affinity of the individual domains. Importantly, even when we calculated the affinity when tethered to the other domain through the linker, this term should not contribute to equation 3.

Remarkably, besides OCT-SOX cooperativity, the POU_S and POU_HD are known to bind cooperatively to the DNA (see [29]). For OCT1, the binding to a consensus binding site couples the affinity of both domains by -1.6 kcal/mol [29]. Although it is unclear if this cooperative effect is present when OCT4 binds to the *UTF1* enhancer, we believe that the co-binding with SOX2 should not affect it, and therefore it is unlikely that it contributes to the OCT4-SOX2 cooperativity.

5 Weighted histogram consistency test

The convergence of the free energy profile obtained from the WHAM procedure depends on the appropriate sampling of the degrees of freedom orthogonal to the chosen reaction coordinate. If the free energy landscape underlying these degrees of freedom features local minima separated by high energy barriers, the sampling might require much longer timescales than typical window lengths. In addition, trajectories where the system remains in a local minimum without visiting any other basins will not show signs of insufficient sampling, making the identification of the problem difficult. Zhu and Hummer [53] have proposed that insufficient sampling can be detected by checking the consistency between the probability distributions calculated from neighboring simulation windows; if different states of the orthogonal coordinates are visited in adjacent windows then inconsistencies between the probability distributions will arise.

Briefly, let us consider two consecutive windows, 1 and 2, with biasing potentials $V_i(r) = k(r - d_i)^2$, where $i = 1, 2$, d_i is the window center, and r the minimal protein-DNA distance. Then, consider a virtual simulation window halfway between them, centered at $d^* = (d_1 + d_2)/2$, with biasing potential $V^*(r) = k(r - d^*)^2$. The corresponding probability distribution $\rho^*(r)$ can be computed from both $\rho_1(r)$ and $\rho_2(r)$ as

$$\rho_i^*(r) = \frac{\rho_i(r)\exp\big([V_i(r) - V_i^*(r)]/k_BT\big)}{\int_{-\infty}^{\infty}\rho_i(r)\exp\big([V_i(r) - V_i^*(r)]/k_BT\big)\mathrm{d}r},\ i \in \{1, 2\} \tag{4}$$

Based on a Kolmogorov-Smirnov test, the inconsistency between the two distributions can be written as

$$\theta_{1,2} = \sqrt{\frac{N_1N_2}{N_1 + N_2}}\max_r\left|\int_{-\infty}^{r}[\rho_1^*(r) - \rho_2^*(r)]\mathrm{d}r\right| \tag{5}$$

where the number of independent samples N_1 and N_2 can be evaluated from the variance of the distribution of r and its average over sub-sets of the corresponding window trajectories (see [53] for details).

Fig. S6 shows the convergence of $\theta_{1,2}$ as a function of the simulation time, for all the simulated complexes. The use of very short simulation windows (0.75 ns) consistently results in small $\theta_{1,2}$ values, because the system does not have time to explore alternative minima of the conformational space of the non-biased degrees of freedom. However, as soon as the sampling time is extended, peaks in the $\theta_{1,2}$ plots appear, showing inconsistencies between the population densities sampled in consecutive windows. These have mostly disappeared for the full window length of 16.5 ns, indicating that the sampling is consistent between consecutive windows. This implies that hysteresis issues that could appear in case of a subdivision of the main dissociation pathway into several branches separated by high free energy barriers are not encountered. There is an expected rise in $\theta_{1,2}$ values with the inter-partner distance, due to the progressive increase of the conformational space volume available as the complex unbinds, together with a gradual decrease of the quality of the sampling achieved with the relatively short simulation time. However, large values of $\theta_{1,2}$ are only reached for separation distances larger than 4.0 Å, at which point the free energy profile has already reached its plateau value.

Therefore, we do not expect major artifacts in the free energy profiles due to insufficient sampling. However, the issue clearly exists, which would prevent the exploration of much larger inter-partner separation distances using the same method.

References

60. Guex N, Peitsch MC. SWISS-MODEL and the Swiss-PdbViewer: an environment for comparative protein modeling. Electrophoresis. 1997 Dec;18(15):2714–2723.

61. Guerois R, Nielsen JE, Serrano L. Predicting changes in the stability of proteins and protein complexes: a study of more than 1000 mutations. J Mol Biol. 2002 Jul;320(2):369–387.

62. Leimkuhler B, Skeel R. Symplectic numerical integrators in constrained hamiltonian-systems. J Comput Phys. 1994;112:117–125.

63. Miyamoto S, Kollman P. SETTLE - an analytical version of the SHAKE and RATTLE algorithm for rigid water models. J Comput Chem. 1992;13:952–962.

64. Essmann U, Perera L, Berkowitz ML, Darden T, Lee H, Pedersen LG. A smooth particle mesh Ewald method. J Chem Phys. 1995;103:8577–8593.

65. Shirts MR, Mobley DL, Chodera JD, Pande VS. Accurate and efficient corrections for missing dispersion interactions in molecular simulations. J Phys Chem B. 2007 Nov;111(45):13052–13063.

66. Zhou HX. The affinity-enhancing roles of flexible linkers in two-domain DNA-binding proteins. Biochemistry. 2001 Dec;40(50):15069–15073.
