# Supplementary material for: Cooperative DNA Recognition Modulated by an Interplay between Protein-Protein Interactions and DNA-Mediated Allostery
Source: PLoS Comput Biol. 2015 Jun 11;11(6):e1004287. doi: 10.1371/journal.pcbi.1004287 (PMC4465831; doi:10.1371/journal.pcbi.1004287)
Supplement: S1 Table — (DOC) [file pcbi.1004287.s002.doc]

**Table S1**: Number of protein-DNA stable contacts seen in the unbiased simulations of the OCT4-*UTF1* and OCT4-SOX2-*UTF1* systems

|  | - SOX2 | | | + SOX2 | | |
| --- | --- | --- | --- | --- | --- | --- |
|  | Backbone | Bases | Total | Backbone | Bases | Total |
| POU_S_ | 139 | 88 | 227 | 123 | 85 | 208 |
| POU_HD_ Tail | 77 | 32 | 109 | 90 | 42 | 132 |
| POU_HD_ Globular | 24 | 4 | 28 | 61 | 6 | 67 |
