# Supplementary material for: Cooperative DNA Recognition Modulated by an Interplay between Protein-Protein Interactions and DNA-Mediated Allostery
Source: PLoS Comput Biol. 2015 Jun 11;11(6):e1004287. doi: 10.1371/journal.pcbi.1004287 (PMC4465831; doi:10.1371/journal.pcbi.1004287)
Supplement: S2 Table — (DOC) [file pcbi.1004287.s003.doc]

**Table S2**: Principle component analysis of the rock/tumble data sets for the POU_S_ domain

|  | λ_1_ | λ_2_ | Angle of first eigenvector with Rock (°) | λ_1_/λ_2_ | Normalized  Area |
| --- | --- | --- | --- | --- | --- |
| With SOX2 | 29.0 | 11.6 | 98.6 | 2.5 | 1 |
| Without SOX2 | 37.7 | 11.6 | 95.4 | 3.2 | 1.14 |
