# Supplementary figures and images for: Cooperative DNA Recognition Modulated by an Interplay between Protein-Protein Interactions and DNA-Mediated Allostery
Source: PLoS Comput Biol. 2015 Jun 11;11(6):e1004287. doi: 10.1371/journal.pcbi.1004287 (PMC4465831; doi:10.1371/journal.pcbi.1004287)

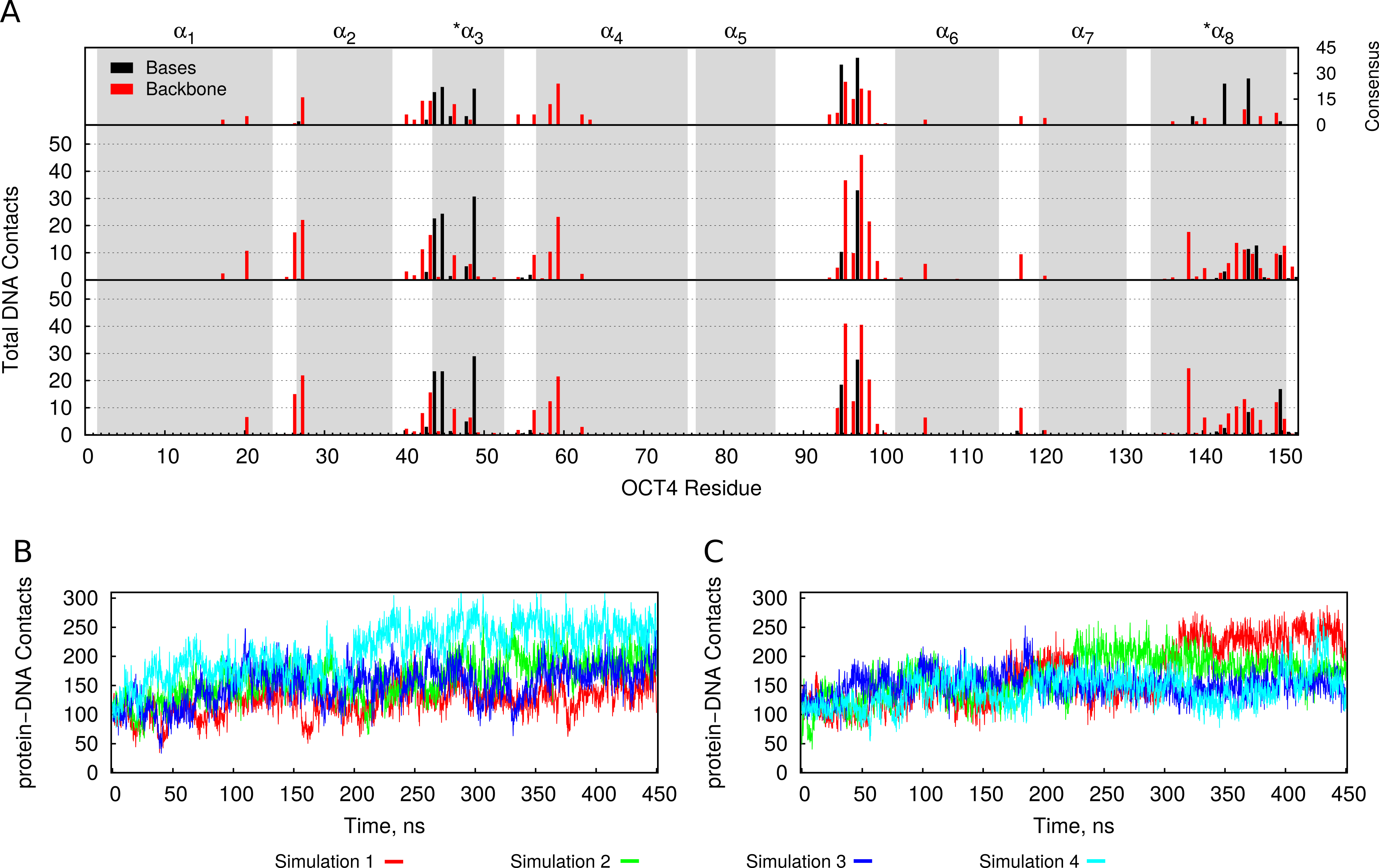

Supplement: S1 Fig — (A) Average number of OCT4-DNA contacts per-residue. The graph on top shows the number of protein-DNA contacts present in a model of the OCT4-SOX2-HOXB1 complex. The gray boxes highlight the 8 helices of OCT4. α1 – α4 correspond to the POUS, while α6 – α8 to the POUHD. (B,C) Evolution of the number of DNA contacts made by the globular region of the POUHD in the absence (B) and presence (C) of SOX2 during the six independent 450 ns-long unbiased simulations. See also Fig 1. (TIF) [file pcbi.1004287.s004.tif]

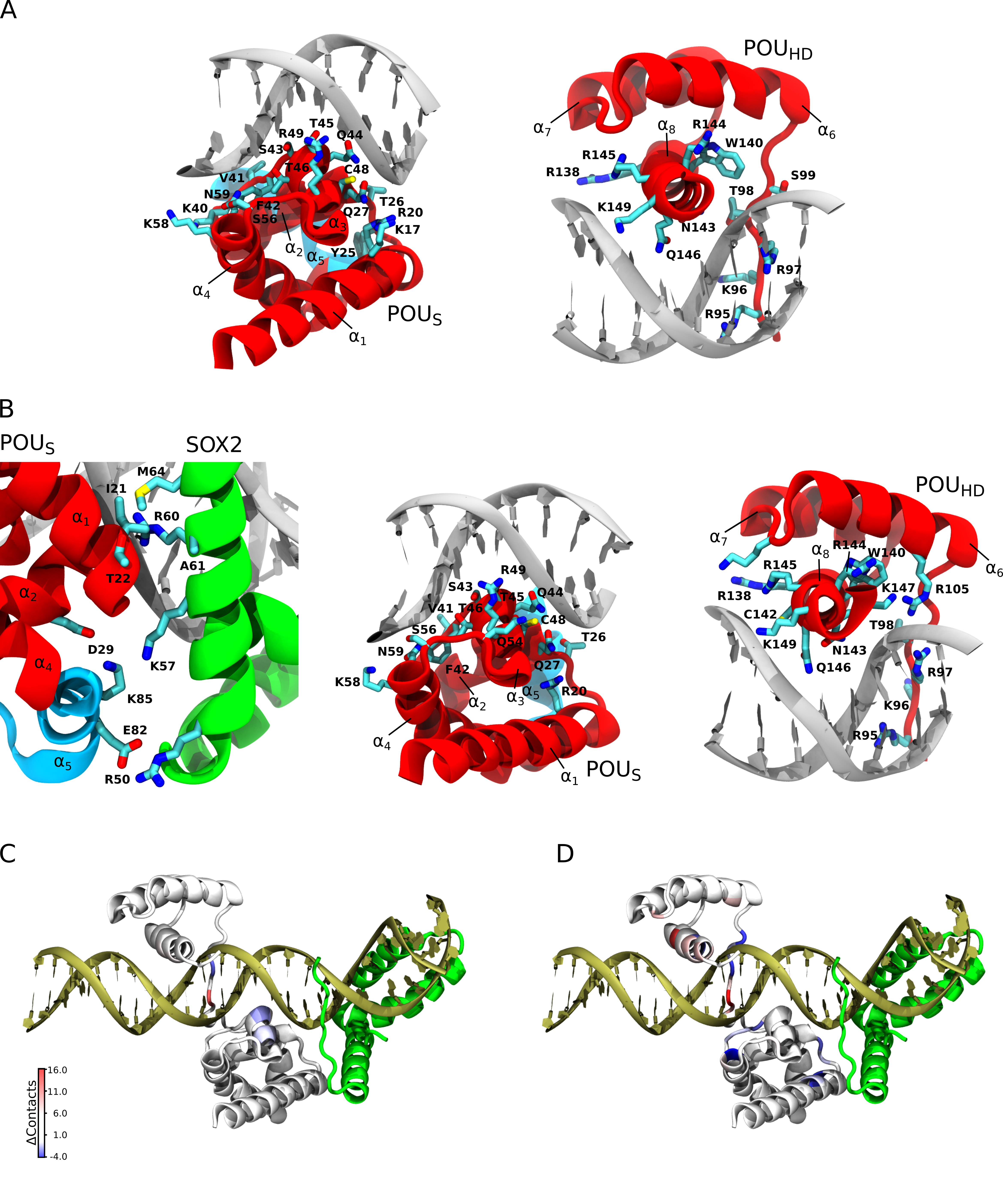

Supplement: S2 Fig — (A,B) Recurrent protein-DNA interactions of the POUS and POUHD in the absence (A) and presence (B middle, right) of SOX2. (B) Protein-protein recurrent contacts at the OCT4-SOX2 interface (left). (C,D) SOX2-induced changes in recurrent protein-DNA interactions with the DNA bases (C) or backbone (D) mapped on the structure of the OCT4-SOX2-UTF1 complex. The color scale shows the difference in recurrent contacts, measured as Q+SOX2—Q−SOX2. See also Fig 1. (TIF) [file pcbi.1004287.s005.tif]

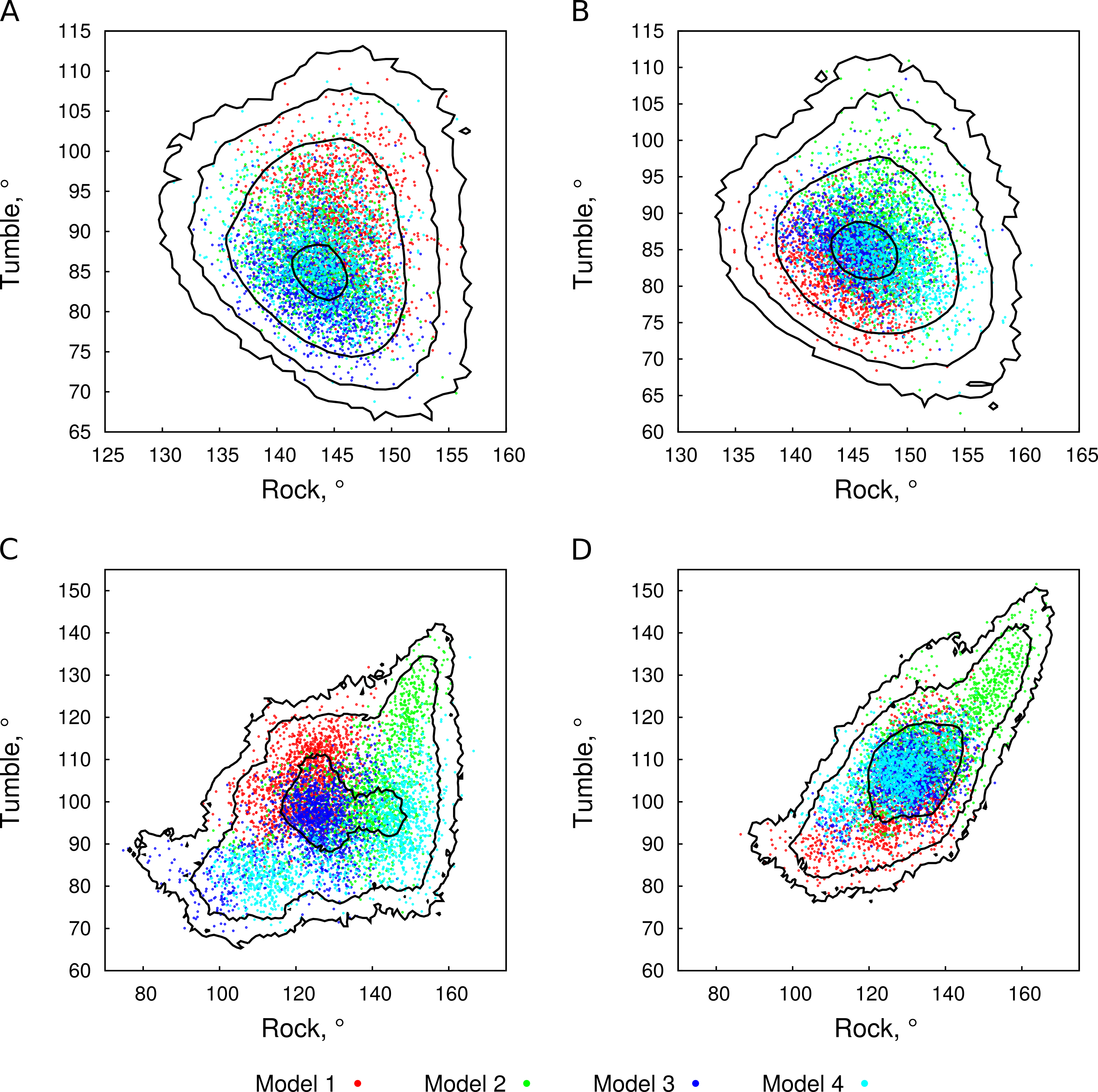

Supplement: S3 Fig — Rock-Tumble measurements in the absence (C,D) or presence (E,F) of SOX2 for the POUS (C,E) and POUHD (D,F). The black lines represent the histograms shown in Fig 2. and are located at 4, 40, 400, 4000 counts. See also Fig 2. (TIF) [file pcbi.1004287.s006.tif]

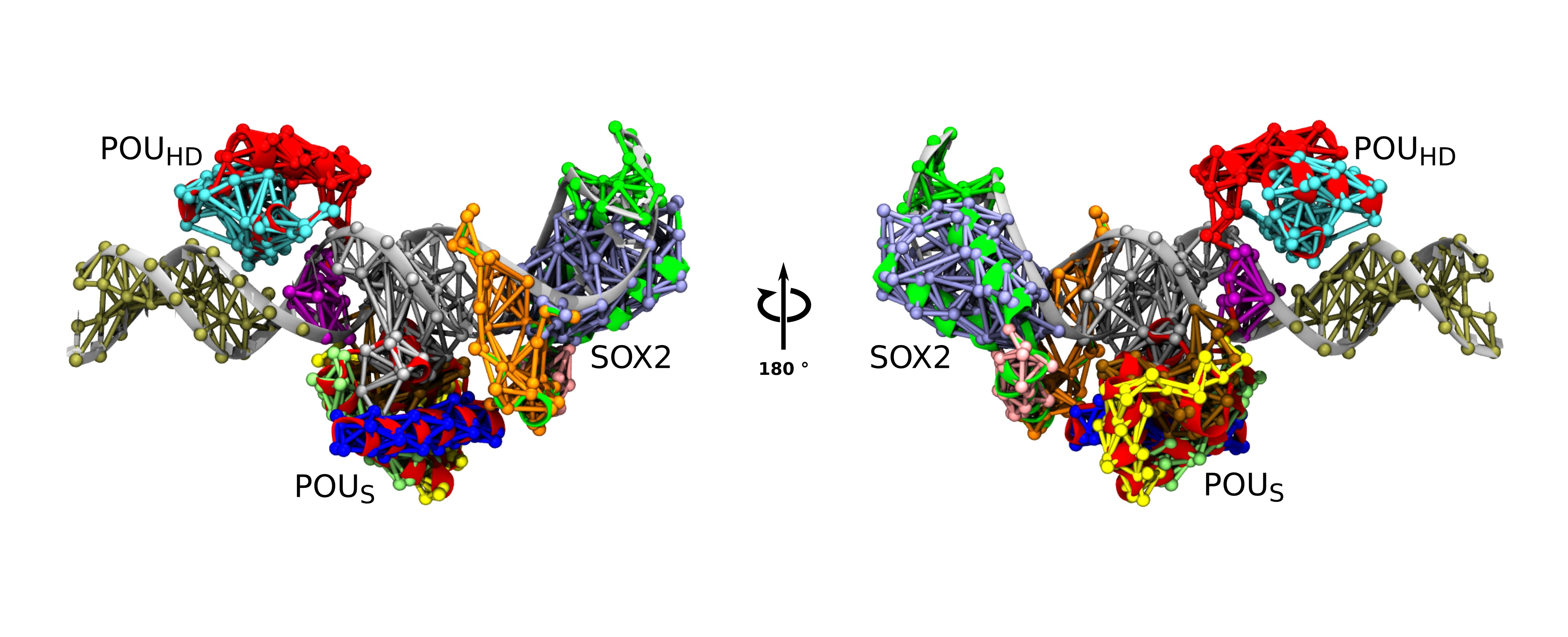

Supplement: S4 Fig — The subnetwork communitites are shown in different colors. View (B) corresponds to view (A) rotated by 180° around the axis shown. See also Fig 3. (TIF) [file pcbi.1004287.s007.tif]

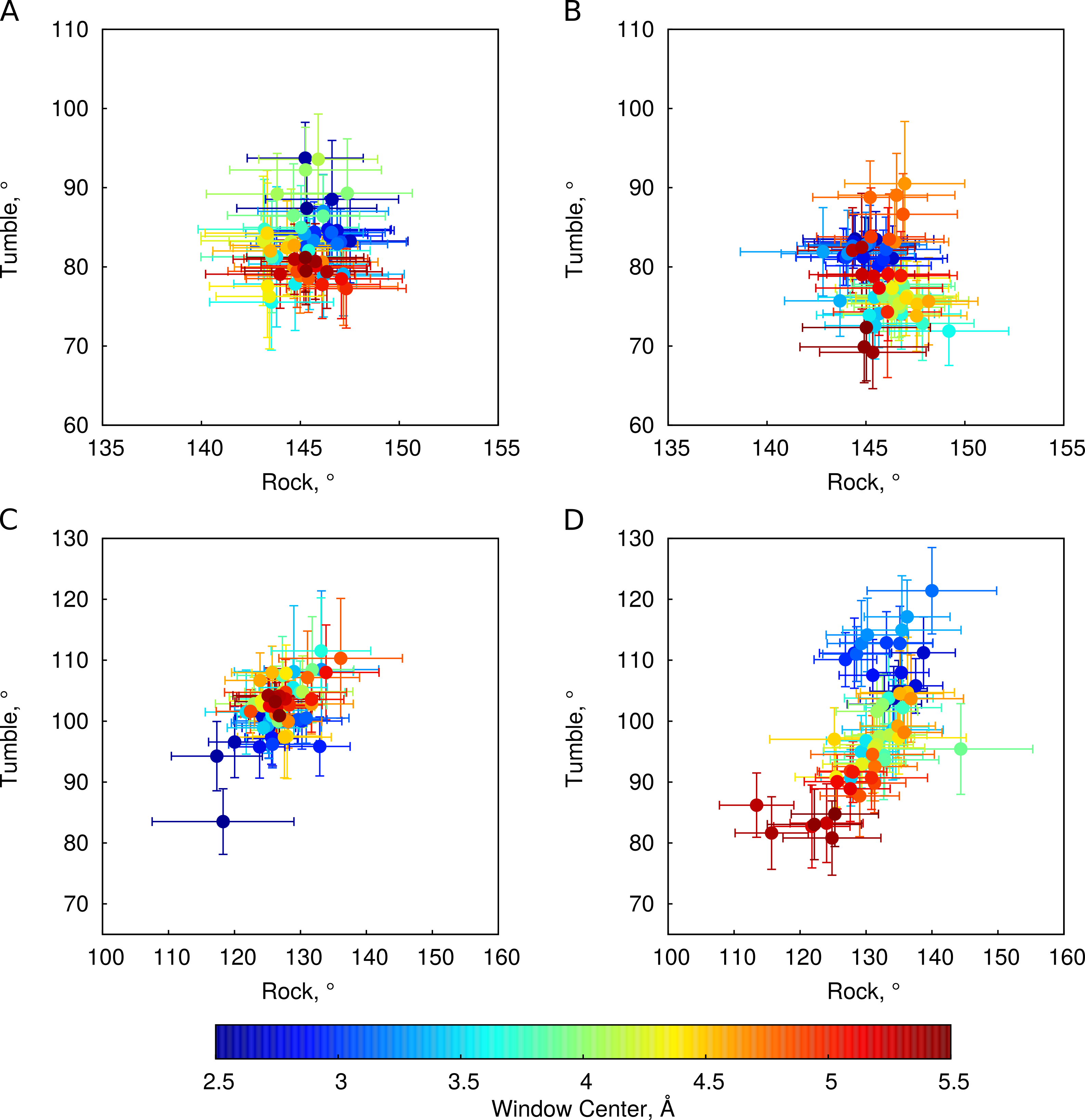

Supplement: S5 Fig — Effect of the unbinding of the POUHD on the orientation of the POUS (A,B). Effect of the unbinding of the POUS on the orientation of the POUHD (C,D). The simulations were performed in the absence (A,C) or presence (B,D) of SOX2. The points show the average and the standard deviation of the Rock and Tumble values from each umbrella window. The color scale represents the protein-DNA separation of the domain being pulled. See also Fig 6. (TIF) [file pcbi.1004287.s008.tif]

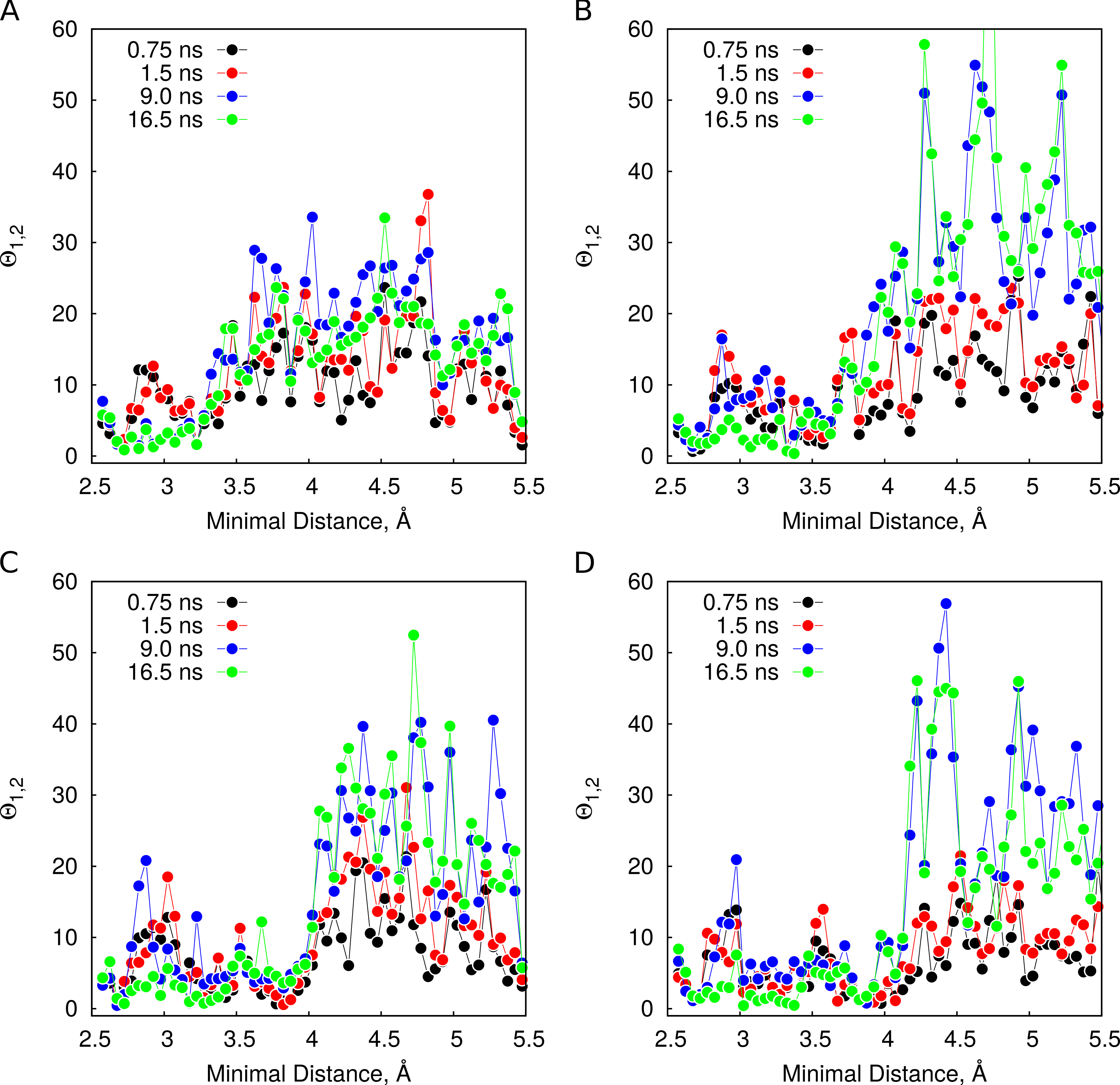

Supplement: S6 Fig — (A,B) POUS in the absence (A) or presence (B) of SOX2. (C,D) POUHD in the absence (C) or presence (D) of SOX2. See also Fig 8. (TIF) [file pcbi.1004287.s009.tif]
